# The Support for Economic Inequality Scale: Development and adjudication

**DOI:** 10.1371/journal.pone.0218685

**Published:** 2019-06-21

**Authors:** Dylan Wiwad, Brett Mercier, Michael D. Maraun, Angela R. Robinson, Paul K. Piff, Lara B. Aknin, Azim F. Shariff

**Affiliations:** 1 Department of Psychology, Simon Fraser University, Burnaby, BC, Canada; 2 School of Social Ecology, University of California, Irvine, Irvine, CA, United States of America; Saint Peter's University, UNITED STATES

## Abstract

Past research has documented myriad pernicious psychological effects of high economic inequality, prompting interest into how people perceive, evaluate, and react to inequality. Here we propose, refine, and validate the Support for Economic Inequality Scale (SEIS)–a novel measure of attitudes towards economic inequality. In Study 1, we distill eighteen items down to five, providing evidence for unidimensionality and reliability. In Study 2, we replicate the scale’s unidimensionality and reliability and demonstrate its validity. In Study 3, we evaluate a United States version of the SEIS. Finally, in Studies 4–5, we demonstrate the SEIS’s convergent and predictive validity, as well as evidence for the SEIS being distinct from other conceptually similar measures. The SEIS is a valid and reliable instrument for assessing perceptions of and reactions to economic inequality and provides a useful tool for researchers investigating the psychological underpinnings of economic inequality.

## Introduction

President Barack Obama recently labeled economic inequality one of the most pressing issues of our time [1]—an assertion that mounting empirical evidence corroborates (e.g. [2–3]). Economic inequality has substantial psychological [4–5] and social consequences [6], such as decreased emotional well-being, reduced physical health, and decreased social trust [7]. Yet, little action is being taken towards addressing these inequalities [2]. Why? One possibility may lie in people’s attitudes toward, and perceptions of, economic inequality––the extent to which inequality is deemed to exist, be problematic, and warrant amelioration. Although measures of attitudes towards economic inequality exist, many of them are single-item measures that, while sometimes useful, suffer from myriad drawbacks. Here, we develop and evaluate a short, reliable, and informative self-report measure of support for economic inequality.

Economic inequality is receiving growing levels of attention in the media and social scientific research, specifically within psychology. For example, within the last two decades alone, there has been an annual increase in the publication of articles mentioning income inequality or economic inequality from 67 articles published in 1993 compared with 937 articles published in 2016 –a 1,298% increase. Interest has risen as researchers aim not just to quantify levels of inequality that exist within a society, but also to understand how it is maintained through perceptions of, and attitudes toward, economic inequality (e.g., [8–11]). Social Psychological researchers have only recently begun exploring the psychological consequences of the daily experience of economic inequality, bridging the gap between abstract economic indicators and psychological experience and perception [7,12].

### Defining “support for economic inequality”

We designed the present scale to measure the degree to which one supports or opposes the current level of economic inequality *as they perceive it*. We specify “as they perceive it” because although people vary in their perceptions of the amount of inequality that exists [13], this scale is not intended to measure those perceptions. Instead, we measure support for whatever level of inequality an individual believes exists.

Importantly, support for economic inequality could be assessed either descriptively (i.e., beliefs about the current state of affairs, for example; [10]) or prescriptively (i.e., beliefs about the way the world should work; [14]). There are myriad descriptive measures of perceptions of economic inequality that aim to assess how much inequality one believes exists. Thus, we do not conceptualize support for economic inequality as a purely descriptive measure (e.g., [10]), or as a purely prescriptive measure. Instead, the SEIS encompasses elements of both descriptive and prescriptive beliefs about economic inequality: survey respondents draw on their own perceptions of economic inequality (i.e., descriptive beliefs) and then report how acceptable they believe this inequality to be (i.e., prescriptive beliefs).

Conceptual work on what constitutes ‘support’ is sparse, especially in a political or ideological context. We adopt a broad definition of support as used in political science. Easton [15] argues that the definition of support as believing something to be “right, valid, just, or authoritative” is an adequate starting point for understanding the construct of “support” in the social sciences. Moreover, this work suggests that the key element of support is the notion of both positive and negative evaluation (i.e., an individual’s support for economic inequality is a result of their evaluations of economic inequality–particularly regarding whether inequality is “right, valid, just, or authoritative”). Thus, we can describe support as a person’s positive/negative evaluations of a construct. In the simplest terms, the extent which a person supports economic inequality reflects the extent which they possess positive or negative evaluations of the current level of economic inequality.

Importantly, we conceptualize support in the context of economic inequality as existing on a spectrum, not a dichotomy. For example, someone may desire that one person holds all the wealth (complete inequality), that some people hold a lot of wealth and some are poor (moderate inequality), or that every single person is equal (no inequality). Therefore, we believe support for economic inequality exists as a continuous latent trait best measured on a spectrum. Furthermore, we consider “support” to be a direct counter to “opposition.” Thus, we consider support for economic inequality as a range from opposition (e.g., negative evaluations; [15]) on the low end to support (e.g., positive evaluations) on the high end.

### Advantages over existing measures

Researchers have not yet validated a multi-item psychological measure assessing support for economic inequality. Numerous indices capture related, but more conceptually broad, constructs (e.g., social dominance orientation, economic system justification, inegalitarianism). Additionally, the measures researchers currently use that directly assess support for economic inequality are all single-item scales. The current scale offers distinct advantages over both the conceptually similar (but not identical) measures, as well as the currently utilized (and conceptually identical) single-item measures of support for economic inequality.

There are several measures in the literature that measure constructs that are conceptually similar, but not identical, to support for economic inequality. For instance, the new egalitarianism subscale of the Social Dominance Orientation scale (e.g., “We should not push for group equality;” [16]), Right Wing Authoritarianism (e.g., “There is no ‘one right way’ to live life; everybody has to create their own way,” [17]), economic system justification (e.g., “If people work hard, they almost always get what they want;” [18]), and beliefs about the sources and consequences of inequality (e.g., “If incomes were more equal, life would be boring because people would all live the same way;” [19]). These previous scales are primarily geared towards measuring and understanding attitudes towards specific group-based differences. For example, a recent paper exploring Social Dominance Orientation demonstrates evidence for an additional sub-construct called “Social Dominance Orientation–Egalitarianism” (SDO-E; [16]). The SDO-E was specifically developed to measure “a preference for systems of group-based inequality that are maintained by an interrelated network of subtle hierarchy-enhancing ideologies and social policies” such as the protestant work ethic, affirmative action, etc. [16]. One might consider the current scale one of these manifestations of hierarchy-enhancing ideologies—the degree to which one is willing to tolerate large income differentials. Thus, the present measure seeks simply to measure the degree to which one supports (or opposes) economic inequality. It is not intended to measure the general belief that some groups should be dominant over others, nor is it intended to measure judgements about the causes and consequences of economic inequality, as is the case with previously established and conceptually similar measures. Importantly, we offer evidence in Study 5 that the SEIS is conceptually distinct from the above-mentioned overlapping measures.

Additionally, there are several existing scales aimed directly at understanding support for economic inequality. However, these pre-existing measures are all single-item questions from large cross-national questionnaires such as the World Values Survey (e.g., “Incomes should be made more equal” versus “We need larger income differences as incentives for individual effort;” [20]), European Values Survey (“Incomes should be made more equal” versus “There should be greater incentives for individual effort;” [21]), or International Social Survey Programme (“Differences in income in <respondent’s country> are too large;” [11,13]). While single-item scales can be useful in some specific contexts (e.g., measuring job satisfaction, [22–23]), their application is limited and there are four distinct advantages the present scale holds over a more face valid and convenient single-item scale.

First, and most crucial, the reliability of a single-item scale cannot directly be assessed with standard methods (e.g., Cronbach’s alpha; [24]). Because of this, it is generally thought that the measurement error of single-item scales is unacceptably high [25]. Second, single-item scales suffer from low sensitivity relative to multi-item scales. For example, one item on a 7-point likert scale has 7 points on which to differentiate all people who answer the question on their underlying level of the construct being measured. On the other hand, a five-item scale that is scored as a sum of five 7-point likert questions has 35 points with which to discriminate respondents. The increased sensitivity that multi-item measures possess allows for a significantly finer-grained and thus more accurate estimate of the construct being measured. Third, because error variance is a component of effect size, if a single-item scale has higher measurement error and lower sensitivity than a multi-item scale, this will result in a decreased effect size relative to a multi-item scale [26]. This decrease in effect size means that single-item scales often have less statistical power than multi-item scales. Lower statistical power and higher measurement error may explain why research using single item scales is less likely to replicate than research using multi-item scales. Fourth, single-item measures cannot be used with modern and complex statistical techniques such as structural equation modeling [27]. Any class of latent variable model (e.g., structural equation models, linear factor analytic models, latent class models, etc) requires more than one item per latent variable in order to make accurate estimates of factor loadings, scores, etc. Specifically, you cannot generate a factor loading or error term for a single indicator on a latent variable—they must be fixed to 1 and 0 respectively. This is a common issue in latent variable modeling known as the single indicator problem. Thus, by creating the present measure we sought to solve this problem and construct a measure of support for economic inequality that can be effectively utilized in latent variable models.

Proper scale development and construct validation procedures are often ignored in social psychology [28], despite the fact that the accuracy of psychological measurement, and by extension the validity of psychological results, is dependent on proper scale development and adjudication. Thus, the present paper offers a short, reliable, and psychometrically sound scale of support for economic inequality that holds several clear conceptual advantages over similar measures, as well as strong methodological advantages over the currently utilized single-item scales.

### Scale development and validation

#### Samples

We collected data from five separate samples on Amazon’s Mechanical Turk (mTurk). Numerous studies demonstrate the usefulness of the mTurk population for data collection (see [29] for a review). Studies show that data collected on mTurk are approximately equivalent to data collected in more traditional populations (e.g., undergraduates) on dimensions such as reliability [30], are more demographically and geographically diverse than data collected in typical university samples, and that many robust psychological effects replicate on mTurk [31]. Moreover, mTurk has been utilized effectively in recent scale development research [32]. These studies were approved by the Research Ethics Board at Simon Fraser University. These studies were approved under application number 2017s0069. Participants electronically provided consent in response to a written consent form.

#### Analytic strategy

We followed Slaney and Maraun’s [33] approach to data-based test analysis using Item Response Theory (IRT) in which we followed five steps to build and evaluate the Support for Economic Inequality Scale (SEIS). First, we generated eighteen potential items capturing the extent to which feelings about economic inequality are positive or negative. Second, we specified the formal structure of the test and identified the corresponding sense of unidimensionality—whether or not the test items measure only the one construct they were designed to measure—and the relevant statistical model to test unidimensionality. Third, we reduced the original eighteen items to a final set of five items, removing those that performed poorly (i.e., did not adequately differentiate between people based on their support for economic inequality). Fourth, we tested the final five items for conformity to the relevant sense of unidimensionality. Lastly, we determined the optimal compositing rule and reliability of the final scale.

### Brief overview of item response theory terminology

Before presenting analyses, we give a brief non-technical overview of Item Response Theory (IRT) and define relevant terminology used throughout the remainder of the paper (for a full review see [34–35]). In contrast to Classical Test Theory (CTT), IRT allows for analysis of how well *each individual item* behaves within the test and allows for a broader assessment of reliability (i.e., determining how reliable the test is for different people, such as those high or low on the trait being measured). IRT accomplishes this improvement on CTT through tools such as Item Characteristic Curves, discrimination, and information functions.

#### Item characteristic curves

**Fig 1:**
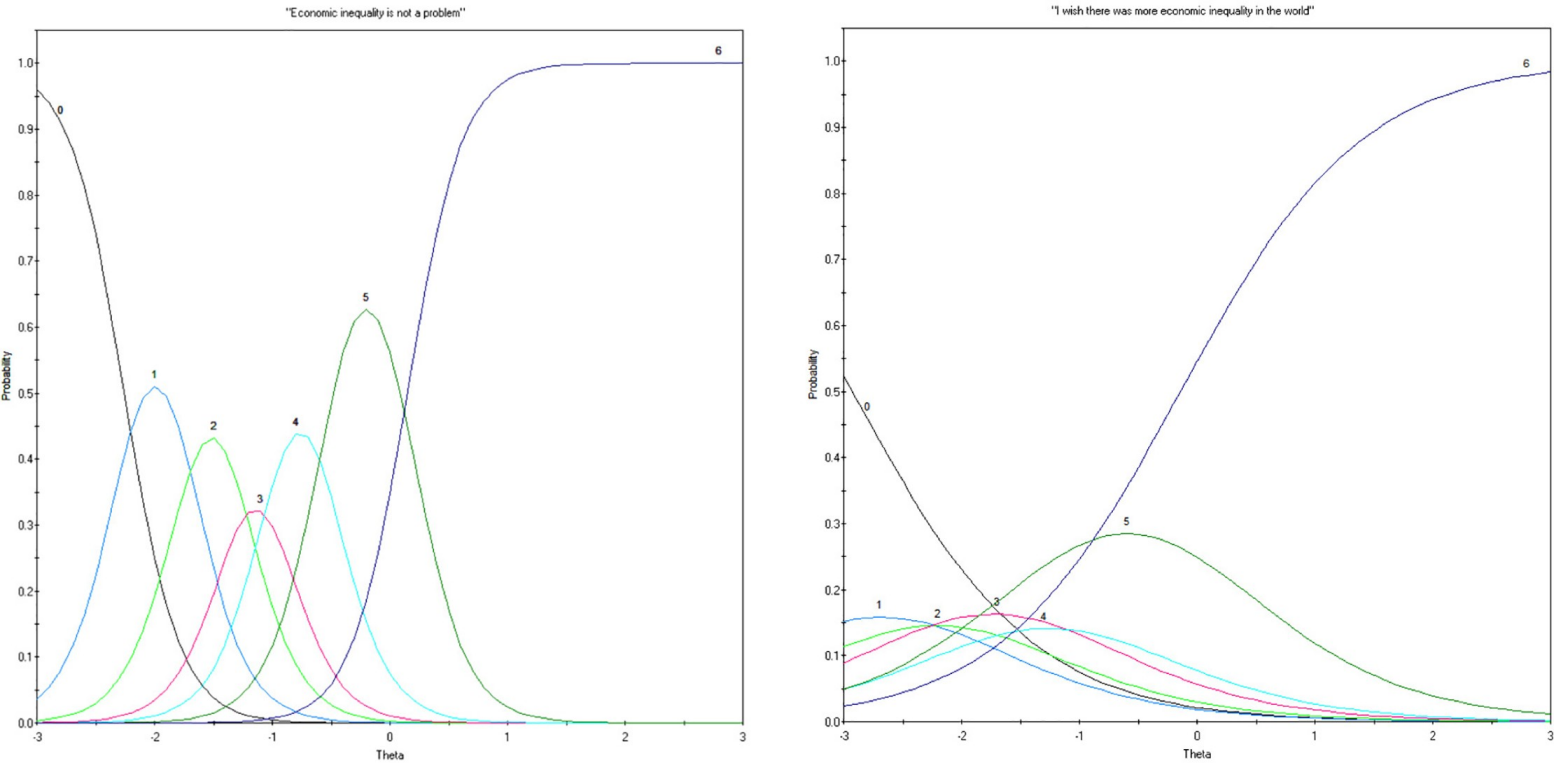
Examples of item characteristic curves from Study 1. (A) A well-defined Item Characteristic Curve; item 10. (b) A poorly-defined Item Characteristic Curve; item 13.

For Likert scale items with more than two response options (i.e. polytomous items), Item Characteristic Curves (ICCs) provide a measure of how well each item differentiates between respondents on the trait being measured by plotting latent “ability” (i.e., *θ*, the underlying level at which a person possesses the trait being measured) along the x-axis, probability along the y-axis, and a curve for each response option. For example, in the present Study 1, item 10 demonstrates a well-defined ICC (Fig 1A). There are clear transition points between each response option, and possessing more extreme support for, or opposition to, inequality (as *θ* moves further from 0 in either direction), leads to increasing probability of selecting the more extreme response. An item that does a poor job of mapping onto the latent underlying construct will have curves that significantly overlap, with messy or disordered thresholds (e.g., the more extreme you are in the latent attitude, the more moderate response options you choose). For example, in the present Study 1 the ICC for item 13 (Fig 1B) shows that the item is functioning dichotomously. Individuals below -1 in *θ* are most likely to choose Strongly Disagree, and individuals above -1 are most likely to choose Strongly Agree. Responding to all middle options on item 13 appears to be essentially chance, and thus uninterpretable.

#### Discrimination

Making a more detailed, numbers-driven assessment of the quality of a polytomous item involves looking at an item’s discrimination [36]. Broadly, discrimination refers to how effectively the item can differentiate between people *at a given θ*. For example, does the difference between 2 and 3 on the scale reflect a small difference in latent support for economic inequality or a large difference? If discrimination is low, the item is poorly differentiating respondents–someone high on the underlying trait is choosing similar response options to someone low on the underlying trait. Discrimination theoretically ranges from 0 to infinity, with higher values (e.g., greater than 1; [37]) indicating that the item adequately differentiates between people at a given *θ*, and is thus more sensitive to changes in the underlying trait.

#### Information

**Fig 2:**
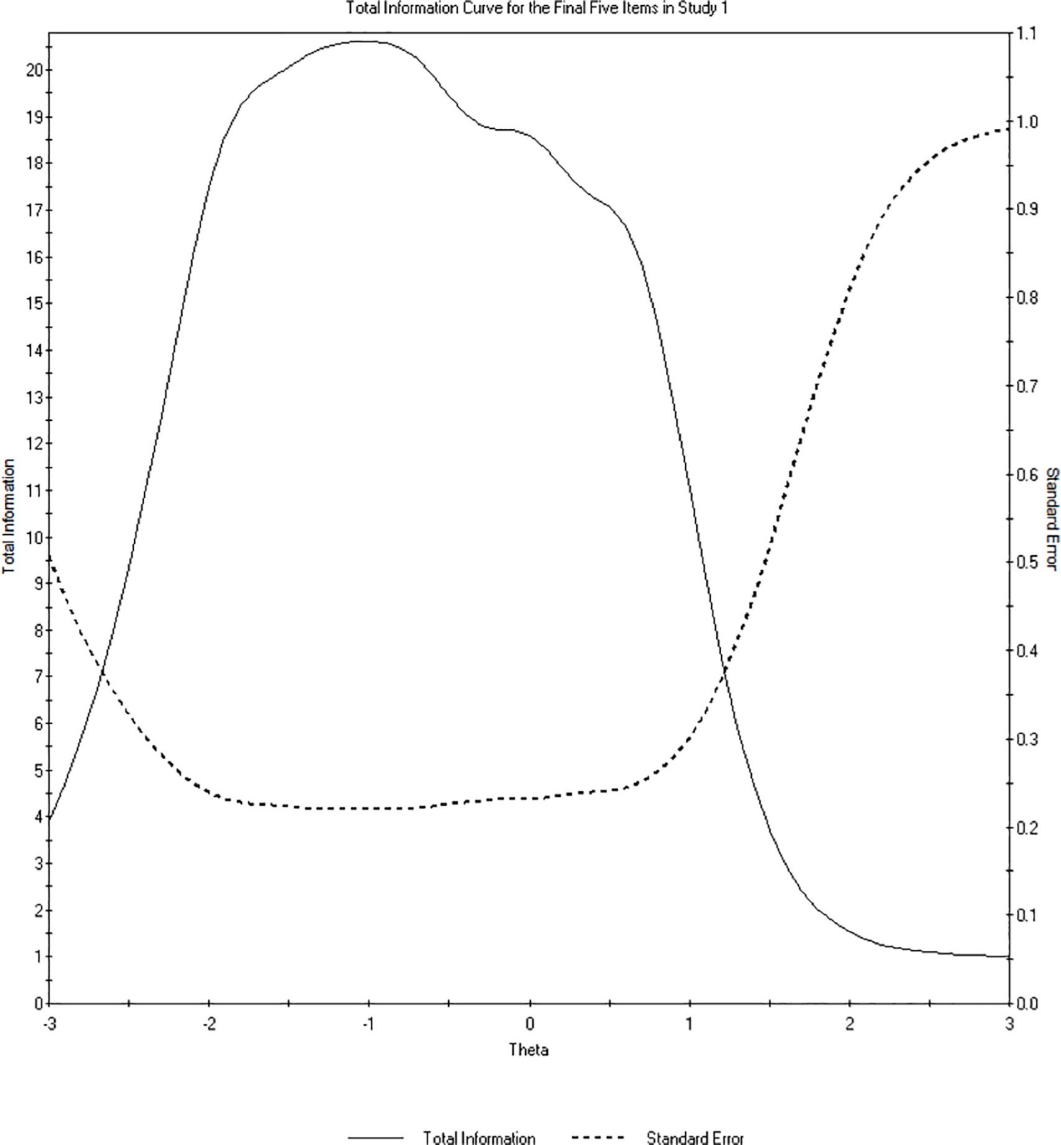
Total information curve for the final five-item scale in Study 1. The SEIS demonstrates high reliability for individuals between -2 and 1 on latent support for economic inequality, θ.

If a test is comprised of highly discriminating items, it provides a great deal of “information” about the underlying latent construct and is thus more sensitive to changes in the underlying trait. Information can be thought of as an assessment of an item or test’s reliability across the *θ* continuum. Information is primarily displayed graphically, with *θ* along the x-axis and total information along the y-axis; a single curve plots the information over *θ* (Fig 2).

### Present studies

With this overview of IRT in mind, we shift to analyses of the SEIS. In Study 1, we employ a step-wise IRT framework [33] in which we develop an initial pool of eighteen items measuring support for (worldwide) economic inequality, remove poorly functioning items, and test the resulting subset for unidimensionality according to Samejima’s Graded Response Model [38]. In Study 2, we confirm the results of Study 1 with a replication. In Study 3, we evaluate a United States version of the SEIS. Finally, in Studies 4–5, we evaluate evidence for the predictive validity of the world-wide version of the SEIS by exploring whether the SEIS predicts inequality mitigating behaviors when compared with other conceptually similar scales. We also demonstrate, via an analysis of conceptual distinctness, that the SEIS is related but sufficiently different from similar pre-existing measures (e.g., Social Dominance Orientation, Right Wing Authoritarianism, etc).

## Study 1

### Data

We collected data from 604 participants (M_age_ = 35.6, 51.8% Female) on mTurk. Participants reported their agreement with the randomly-ordered eighteen item SEIS (see Table 1). Afterward, participants reported their age, gender, and political ideology. We coded the items such that endorsement of higher response options indicated more support for economic inequality.

**Table 1:** Original 18 items, with their descriptive statistics from Study 1. (R) denotes item is reverse coded. Descriptive statistics were calculated after each relevant item was reverse scored.

	Item content	Mean	SD	Skewness	Kurtosis
1.	Economic inequality is one of the biggest problems in today’s world. **(R)**	2.20	1.84	0.56	-0.82
2.	Economic inequality is not a big problem in the world	1.51	1.65	1.00	-0.05
**3.**	**The negative consequences of economic inequality have been largely exaggerated.**	**1.66**	**1.67**	**0.91**	**-0.10**
4.	Economic inequality is mostly caused by different levels of individual effort.	2.24	1.72	0.30	-0.96
**5.**	**Economic inequality is causing many of the world’s problems. (R)**	**1.90**	**1.64**	**0.76**	**-0.18**
6.	Economic inequality is inherently unfair. **(R)**	1.96	1.72	0.70	-0.41
7.	I am not bothered by the current level of economic inequality in the world.	1.77	1.83	0.87	-0.36
**8.**	**I am very disturbed by the amount of economic inequality in the world today. (R)**	**1.87**	**1.74**	**0.77**	**-0.37**
9.	There are much bigger problems in the world than economic inequality.	3.02	1.82	-0.11	-0.98
**10.**	**Economic inequality is not a problem.**	**1.25**	**1.50**	**1.20**	**0.60**
11.	There are some positive benefits that result from economic inequality.	1.34	1.49	0.98	-0.05
12.	Overall, economic inequality is good for the world.	1.44	1.55	0.98	0.12
13.	I wish there was more economic inequality in the world.	1.23	1.71	1.40	0.93
14.	Economic inequality is fair.	1.63	1.70	0.91	-0.12
15.	I am very concerned about the current level of economic inequality in the world. **(R)**	1.93	1.72	0.78	-0.27
16.	If I could, I would make the world a more equal place. **(R)**	1.44	1.49	1.12	0.89
17.	Economic inequality does not lead to anything good.	2.14	1.72	0.33	-1.02
**18.**	**We need to do everything possible to reduce economic inequality in the world today. (R)**	**1.87**	**1.64**	**0.81**	**-0.04**

*Note*. Bolded items are the final five-item “Support for Economic Inequality” scale, as determined in Study 1

### Step 1: Original item generation

Our goal was to begin with an expansive list of items that we could empirically narrow down to the best functioning subset. To this end, we generated an initial set of eighteen items using an inductive approach ([39], see Table 1 for items).

Descriptive analyses of the original eighteen items showed that most items yielded a significant positive skew, indicating that the majority of our sample endorsed response options consistent with quite strong opposition to inequality. One explanation for this skew is that our participants were generally politically liberal (72% of participants fell on or above the midpoint on a 1 (Conservative) to 9 (Liberal) scale; *M* = 5.70, SD = 2.50), and may hold relatively extreme opposition to economic equality. Additionally, non-normality of the data is not uncommon in psychology [40], and the observed positive skew may indicate that most people in the United States have a negative view of economic inequality. Some of the issues regarding skewness of the items will be addressed further in the differential item functioning analyses.

### Step 2: Theoretical structure of the test and relevant sense of unidimensionality

The statistical techniques used to evaluate a test, and assess for unidimensionality, are inherently linked to the Theoretical Structure (TS) of the scale. There are, at minimum, five components that require specification in the TS: The underlying distribution of the latent construct, the item response formats, the number of latent attributes the test is designed to measure, the form of the item/attribute regressions, and whether or not the items are error-laden [33]. We chose to administer the items as 7-point polytomous items (i.e., a Likert scale ranging from 1 = Strongly Disagree to 7 = Strongly Agree) whereby people with a stronger support for, or opposition to, economic inequality should be more likely to endorse response options increasingly further above/below the midpoint, respectively. We outlined the formal structure of the present Support for Economic Inequality scale to be:

TS{Co,7PL,1,7OC,EIV}

Thus, this is a test for which a set of 7-point Likert (7PL) items are designed as error-laden (EIV) indicators of a single underlying attribute (1; support for economic inequality), which continuously varies (Co) in degree over a population. Moreover, for any given item, the relationship between the item and the underlying attribute is conceived of as a set of seven item/attribute regressions in which the probability of endorsing any given category varies with the degree to which the individual possesses the underlying attribute (7-point ordered categorical (7OC); [33]). For instance, for someone who holds extremely strong support for economic inequality, the probability they will choose “7” is higher than the probability they will choose “6,” which is higher than the probability they will choose “5,” and so on.

From the TS, we can determine the relevant quantitative characterization of the test. The quantitative characterization describes how our test is said to behave mathematically if it is unidimensional according to our TS. When we understand how a unidimensional test should behave (i.e., the expected pattern of responses) according to our TS, we can then choose the appropriate statistical model with which to test for unidimensionality. Following the recommendations in Slaney and Maraun [33], for our specified TS, we expect our test to be unidimensional in the sense of Samejima’s Graded Response Model (GRM; [38]). Samejima’s GRM claims that the probability of an individual with a certain level of the underlying trait choosing a given response option is equal to the probability that they will choose that particular option (or any lower option), minus the probability they will choose the option that is one higher (see [41] for a more technical discussion of how these probabilities are determined and calculated). For example, the probability of someone with moderately positive support for economic inequality choosing option 5 (i.e., Slightly Agree) to a particular question is equal to the cumulative probability of them choosing options 1 through 5 minus the probability of them choosing option 6 (i.e., “Agree”).

Utilizing Samejima’s GRM, we do not expect our test to conform to the typical linear factor analytic assessment of unidimensionality; that is, unidimensionality in this case does not mean a set of items with high factor loadings onto one latent “common factor.” Instead, the relevant test for unidimensionality is a set of quasi chi-square statistics testing the null hypothesis that the observed response probabilities for each item are in line with the expected probability structure laid out by Samejima’s GRM [33, 41–42]. Retaining the null hypothesis suggests that the observed response probabilities for each item are in line with the response probabilities we would expect given Samejima’s GRM, and thus the set of items are unidimensional.

### Step 3: Initial item reduction/selection

We conducted all of our IRT individual item analyses using the ltm package in R [43–44]. All decisions were data-driven; we were unaware of the content of items during analyses. We based our decisions to remove items on several factors: we evaluated each item individually using its Item Characteristic Curve (ICCs), the relative proportion of information it contributed to the scale’s overall information, and its discrimination.

We identified a subset of seven items from the original 18 that had the highest discrimination, contributed the most to the total overall scale information, and had the most clearly defined ICCs (S1–S18 Figs): 2, 3, 5, 8, 10, 15, and 18. However, there were some content and threshold redundancies among these seven items. Particularly, items 8 and 15 revealed nearly identical thresholds, meaning these items were redundant. This redundancy is also apparent in content: “I am very disturbed by the amount of inequality in the world today” and “I am very concerned about the current level of economic inequality in the world.” As such we kept only item 8, as it had slightly higher discrimination. The same was true of items 2 and 5. Because these items were nearly identical, we arbitrarily chose to keep item 5. This left a final five-item scale containing items 3, 5, 8, 10, and 18 (bolded in Table 1).

Because IRT parameters are calculated relative to the complete set of items included in the scale, we re-evaluated the individual item functioning for the set of five items. This analysis demonstrates that the final set of five items function better than the original set of 18 items (S1 and S2 Tables). Discrimination values are higher, each item contributes roughly equivalent information to the scale total, and the ICCs are uniformly sharper and more cleanly defined. Following this identification of five apparently acceptably functioning items, we turned to an assessment of unidimensionality.

### Step 4: Assessment of unidimensionality

To test the hypothesis of unidimensionality we utilized quasi chi-square statistics calculated with the IRTPro software [45], one for each of the five test items. These statistics quantify the difference between observed response probabilities and those expected under Samejima’s GRM [37]. A problem with the chi-square approach, however, is that large sample sizes result in over-powered chi-square tests that reject the null hypothesis for very small deviations from the expected probabilities, erroneously suggesting that Samejima’s GRM is a poor fit and the items are not unidimensional. Thus, we considered the hypothesis of unidimensionality to be rejected if a quasi chi-square statistic was greater than three times its degrees of freedom [46].

Because all five statistics were less than three times their degrees of freedom (Table 2), the hypothesis of unidimensionality was retained (i.e., we infered that the five items measure a common underlying trait, presumptively support for economic inequality). Next, we determined the optimal compositing rule and assessed the point-estimated reliability of the resulting five items.

**Table 2:** Goodness-of-fit Chi-Square tests for the five-item scale in Study 1.

Item	Chi-square	df	p-value	Chi-square/df
3	84.37	69	.10	1.22
5	84.91	71	.12	1.20
8	74.61	64	.17	1.17
10	89.32	69	.05	1.29
18	95.29	71	.03	1.34

### Step 5: Model implied compositing rule and reliability assessment

**Fig 3:**
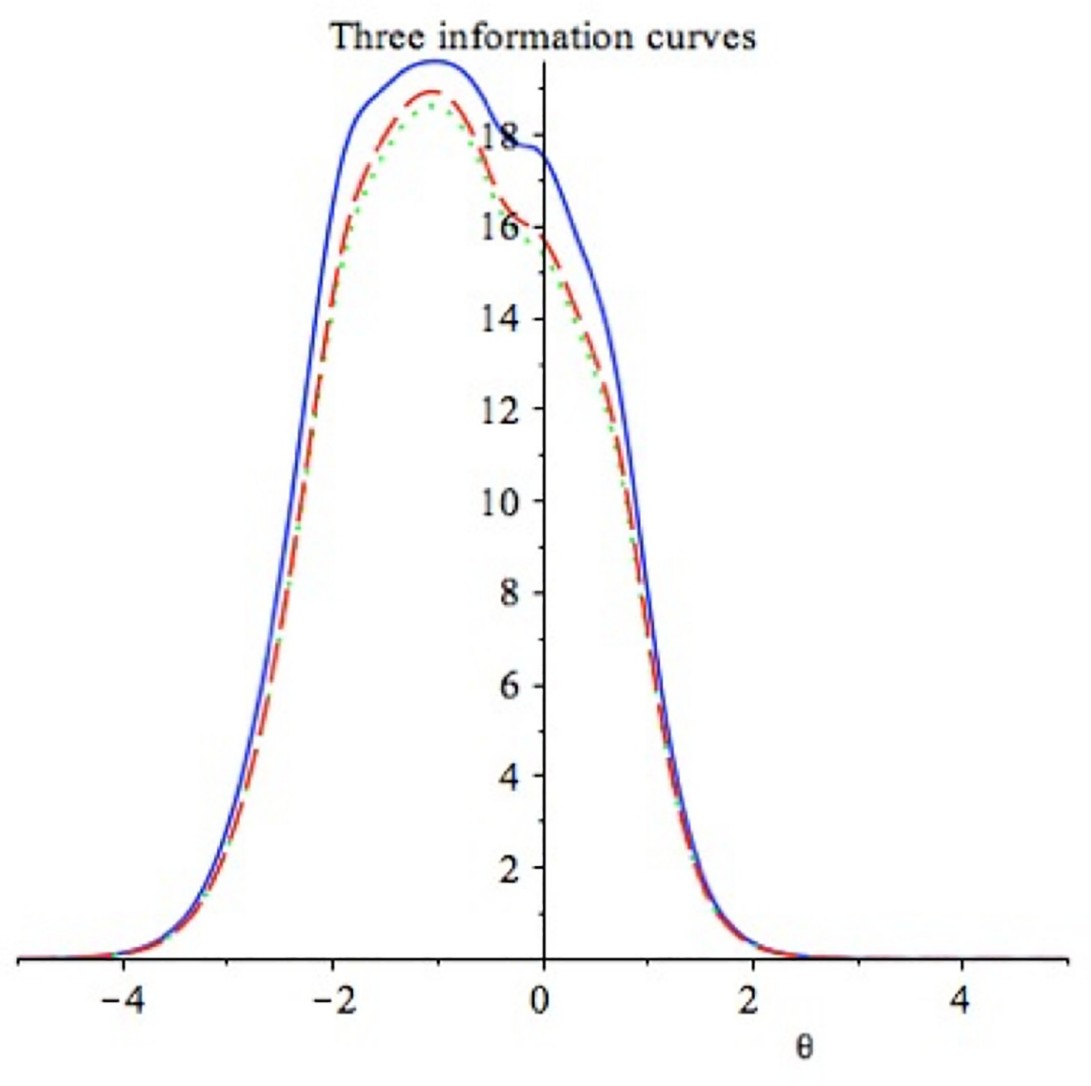
Study 1 information function for the three candidate compositing rules. The solid line is the Maximum Likelihood estimated theoretical maximum information, the dashed line is the *a*_*j*_ weighted composite information, and the dotted line is the unit-weighted composite information.

We computed information functions (Fig 3) for two common candidate compositing rules for psychological scales (See https://osf.io/cmzye/ for Maple [47] worksheet containing the calculations): (1) a linear weighted estimator (using the *a*_*j*_ slope parameter as the weight for each item), and (2) a unit-weighted estimator (simply adding the unweighted items together). We graphically compared the information functions of the composited scale calculated under each of these two compositing rules with the non-linear maximum likelihood estimator of *θ* (the theoretical information maximum). Fig 3 shows that neither compositing rule creates a scale that provides as much information about the underlying trait as the theoretical maximum. However, the two composited versions of the scale appear to provide identical information (i.e., the curves almost completely overlap). Given that the slope and unit weighted composited performed nearly identically, and unit weights are simpler to work with (i.e., participants responses can be averaged without weighting each item by its factor loading, *a*_*j*_), we decided to nominate unit weighting as the ideal compositing rule for the SEIS. Thus, researchers using the SEIS should not weight the items by factor scores but should compute the composite as a sum or mean for each participant. The reliability of the unit-weighted linear composite for the final five-item scale was .94 (See Maple worksheet at https://osf.io/cmzye/ for reliability and information curve calculations).

### Discussion

In Study 1 we generated an initial set of eighteen items measuring support for economic inequality and then distilled these items to the best functioning five items (See Table 1). Finally, we determined that this set of five items is unidimensional (measures one underlying construct), should be composited using a unit-weighted sum or mean calculation, and demonstrates a high degree of point-estimated reliability. While Study 1 provides evidence for a psychometrically sound and reliable measure of support for economic inequality, we did not explore questions of convergent and discriminant validity. Thus, in Study 2 we aimed to replicate the findings of Study 1 and provide additional evidence for convergent validity of the SEIS.

## Study 2

In Study 2 we aimed to replicate and extend the findings of Study 1 with the final five-item measure. To this end, we collected a separate sample from mTurk and ran the same individual item analyses and tests of unidimensionality as Study 1. Additionally, we explored convergent validity by including measures of just world beliefs [48], support for redistribution [20], wealth guilt [49], as well as face-valid measures of perceived level of inequality, perceived growth in inequality, belief that inequality is unchangeable, perceived warmth and competence of people in poverty (adapted from [50]), empathy [51], prosocial tendencies [52], income, and political ideology. The three scales measuring perceived level and growth of inequality as well as the belief that inequality is unchangeable were created in-house as short, face-valid measures specifically for this paper.

### Data

We collected data from 657 participants (M_age_ = 23.8, 56.6% Female, Political Party identification: 40.5% Democrat, 27.9% Independent, 27.1% Republican, 4.6% Other, *Median*_*income*_ *=* $40,000 - $49,999) on mTurk. Participants completed the five-item SEIS (items presented in random order). Afterward, participants filled out, in random order, measures of just world beliefs [48], support for redistribution [20], wealth guilt [49], perceived level of inequality (e.g., “Overall, the world is a fairly equal place”), perceived growth in inequality (e.g., “Economic inequality in the world is growing faster than ever before”), belief that inequality is unchangeable (e.g., “Economic inequality cannot be prevented”), perceived warmth and competence of people in poverty (adapted from [50]), empathy [51], prosocial tendencies [52], income, political ideology, and demographics. Identical to Study 1, we coded responses on the SEIS such that endorsement of higher response options indicated more support for economic inequality. All materials are available on https://osf.io/cmzye/.

### Individual item evaluation

Similar to Study 1, we assessed the ICCs, information, and discrimination values for the scale. Replicating Study 1, all items demonstrated generally sharp and clearly defined ICCs, indicating that they function with a high degree of discrimination and are effective at differentiating between people on their underlying support for economic inequality. All discrimination values were above 3, all items contributed a high degree of information (> 9) to the total scale (Information = 62.57; S3 Table). These analyses confirm the selection of the final five items, which comprise a small but highly differentiating and useful set of items.

### Assessment of unidimensionality

Consistent with Study 1, we tested the hypothesis of unidimensionality with a quasi chi-square statistic for each of the five items. As all chi-square statistics were less than 3 times their degrees of freedom (S4 Table), we replicate Study 1 and retain the hypothesis that the set of five items are unidimensional.

### Model implied compositing rule and reliability assessment

We computed information functions (S19 Fig) for the same candidate compositing rules as Study 1. Replicating Study 1, we found that the two candidate compositing rules performed equally well, confirming our decision to nominate unit weighting as the ideal compositing rule for the SEIS. The reliability of the unit-weighted linear composite for the five-item scale was .94.

### Convergent and discriminant validity

To assess convergent and discriminant validity we ran a series of correlations between the SEIS and various measures of related psychological constructs. Demonstrating evidence for convergent validity, we found that more positive support for economic inequality was related to higher: political conservatism, social conservatism, economic conservatism, belief that inequality is unchangeable, belief in a just world, and income (see Table 3). Additionally, we found that more positive support for economic inequality was related to decreased: perceived inequality, support for redistribution, wealth guilt, belief that the poor are competent, belief that the poor are warm, empathy, and prosocial tendencies (see Table 3). Demonstrating evidence for discriminant validity, we found no relationship between support for economic inequality and gender (*r* = -.07, *p* = .07) or age (*r* = -.04, *p* = .31).

**Table 3:** Correlations between all scales assessing convergent validity in Study 2.

	*1*.	*2*.	*3*.	*4*.	*5*.	*6*.	*7*.	*8*.	*9*.	*10*.	*11*.	*12*.	*13*.
1. SEIS	—												
2. GC	.58***	—											
3. SC	.52***	.89***	—										
4. EC	.57***	.87***	.75***	—									
5. IU	.72***	.49***	.45***	.51***	—								
6. PI	-.56***	-.32***	-.31***	-.30***	.40***	—							
7. SR	-.78***	-.52***	-.47***	-.54***	-.68***	-.41***	—						
8. WG	-.26***	-.21***	-.18***	-.25***	-.18***	.05	.26***	—					
9. Comp	-.23***	-.11**	-.08	-.18***	-.21***	.02	.22***	.26***	—				
10. Warm	-.25***	-.07	-.08*	-.14***	-.24***	-.03	.24***	.18***	.71***	—			
11. Emp	-.28***	-.11**	-.12**	-.12**	-.25***	-.39***	.24***	-.04	-.03	.13***	—		
12. PT	-.20***	-.05	-.06	-.10*	-.16***	-.10**	.17***	.19***	.23***	.30***	.40***	—	
13. BJW	.33***	.29***	.29***	.28***	.31***	.36***	-.26***	.01	-.01	.00	.01	.08*	—
14. Inc	.11**	.09*	.06	.13***	.16***	.01	-.09*	.07	-.15***	-.16***	.02	.03	.09*

*Note*. SEIS = Support for Economic Inequality; GC = General Conservatism; SC = Social Conservatism; EC = Economic Conservatism; IU = Belief that Inequality is Unfixable; PI = Perceived Inequality; SR = Support for Redistribution; WG = Wealth Guilt; Comp = Perceptions of the poor as competent; Warm = Perceptions of the poor as warm; Emp = Empathy; PT = Prosocial Tendencies; BJW = Belief in a Just World; Inc = Income.

* = *p* < .05

** = *p* < .01

*** = *p* < .001

### Discussion

Study 2 replicated Study 1 by demonstrating that the SEIS is comprised of a small set of effective, unidimensional, and reliable items. Additionally, Study 2 provides evidence for convergent and discriminant validity. However, Studies 1–2 measure support for *worldwide* economic inequality. Because many researchers are concerned with more localized inequality (e.g., country, state, or county), and because much of this research is being conducted in the United States, we sought to extend our findings by testing a U.S. version of the SEIS.

## Study 3

In Study 3 we evaluated a U.S. version of the SEIS. To do this, we took the original five-item scale and replaced every instance of the word “world” with “United States.” For example, “economic inequality is causing many of the world’s problems” became “economic inequality is causing many of the United States’ problems.” We administered this scale on mTurk and ran the same individual item analyses and tests of unidimensionality used in Studies 1–2. Additionally, we utilized the measures from Study 2 to explore convergent and discriminant validity, as well as additional measures of free will, over-claiming, and socially desirable responding. Over-claiming measures self-enhancement through participants’ willingness to claim they possess knowledge they actually do not. Over-claiming was measured by the extent to which a person claimed to be knowledgeable in non-existent subjects (e.g., Plates of Parallax). We included these measures to examine whether the SEIS is susceptible to social desirability or self-enhancement effects.

### Data

We collected data from 619 participants (M_age_ = 36.01, 52.7% Female, Political Party identification: 43.6% Democrat, 32.6% Independent, 19.7% Republican, 3.9% Other, *Median*_*income*_ *=* $50,000 - $59,999) on mTurk. Participants first completed the five-item U.S. version of the SEIS (items presented in random order). Afterward, participants filled out, in random order, the measures of convergent and discriminant validity from Study 2, as well as measures of free will [53], over-claiming [54], socially desirable responding [55], and demographics. Identical to Studies 1–2, items were coded such that higher responses indicated more support for economic inequality. Materials available at https://osf.io/cmzye/.

### Individual item evaluation

Consistent with Studies 1–2, items demonstrated generally sharp and clearly defined ICCs, indicating that they function with a high degree of discrimination and are effective at differentiating between people on their underlying support for economic inequality. All discrimination values were above 3, all items contributed a high degree of information (> 9) to the total scale (Information = 62.76), and all ICCs were similar in profile to Study 2 (S20–S24 Figs). The results of these analyses demonstrate that the U.S. version of our scale functions similarly to the more general worldwide version, and that the five items comprise a highly discerning and useful set of items.

### Assessment of unidimensionality

Following Studies 1–2, we computed quasi chi-square statistics for each of the five items (S5 Table). Replicating Studies 1–2, all quasi chi-square statistics were less than three times their degrees of freedom. Thus, we again retain the hypothesis that the items are unidimensional.

### Model implied compositing rule and reliability assessment

We computed information functions (S25 Fig) for the same candidate compositing rules as Studies 1–2 (See https://osf.io/cmzye/ for Maple worksheet containing calculations). As in Studies 1–2, two candidate compositing rules had similar performance, although the unit weighted composite performed only slightly worse than the *a*_*j*_ weighted composite. While the unit-weighted version appears to give slightly less information, we do not believe the loss of information is enough to justify significantly complicating the compositing rule. The reliability of the unit-weighted linear composite for the five-item United States version of the scale was .94.

### Convergent and discriminant validity

To assess convergent and discriminant validity we ran a series of correlations between the U.S. version of the SEIS and the same measures of political attitudes and psychological constructs as Study 2, plus additional measures of free will, over-claiming, and socially desirable responding. Similar to Study 2, as evidence for convergent validity we found that more support for economic inequality was related to higher: general conservatism, social conservatism, economic conservatism, belief that inequality is unfixable, belief in a just world, free will, and income (S6 Table). Additionally, we found that more support for economic inequality was related to decreased: perceived inequality, support for redistribution, wealth guilt, belief that the poor are competent, belief that the poor are warm, empathy, and prosocial tendencies. Additionally, as evidence of discriminant validity, we found no relationship between support for economic inequality and gender (*r* = -.06, *p* = .16) or age (*r* = -.03, *p* = .51). Support for economic inequality was uncorrelated with over-claiming (*r* = -.04, *p* = .38) and socially desirable responding (*r* = .00, *p* = .98).

To test the usefulness of the SEIS, we explored whether it can predict behavior relevant to economic inequality. Specifically, Studies 4–5 test whether the SEIS can predict signing a petition and donating to a group related to inequality.

## Study 4

### Participants

We collected data from 117 participants (M_age_ = 35.31, 59.0% Female, *M*_*pol*. *id*._ = 3.28 (1 = Very Liberal to 7 = Very Conservative), *Median*_*income*_ *=* $35,001 - $50,000) on mTurk. These data were part of a larger study in which there was a manipulation. Participants either read an article about (a) hardworking poor people in America, (b) about the poor in America more broadly, or (c) about gun violence in America. These articles can be found on the OSF page for this paper. Condition assignment did not impact scores on the SEIS, *F*(1,115) = 1.012, *p* = .32.

### Procedure

Participants were presented with a petition for a 39% increase in the minimum wage in the U.S. from $7.25/hour to $10.10/hour (See https://osf.io/cmzye/ for petition). After, participants were asked “to what extent do you agree with the content of the petition” on a 1 (Strongly disagree) to 7 (Strongly agree) scale and were given the opportunity to sign the petition by entering their first and last name, email address, and zip code. Next, participants filled out the worldwide version of the SEIS and demographics (age, gender, education level, household income, political ideology, and the level of inequality present in the area in which they grew up; see https://osf.io/cmzye/ for questionnaire).

If the SEIS has adequate predictive validity, higher support for economic inequality should predict (a) decreased agreement with the petition and (b) decreased likelihood of signing the petition. We predicted these relationships remain when controlling for age, gender, education, household income, political ideology, and the level of inequality in a participant’s childhood neighborhood.

### Results

A linear regression indicated that higher support for economic inequality was associated with lower agreement with the petition. Importantly, this relationship remained when controlling variables that might influence agreement with increasing minimum wage (Table 4). In addition, a logistic regression demonstrated that higher support for economic inequality was associated with lower likelihood of signing the petition. Once again, this relationship remained when controlling for relevant demographics (Table 5).

**Table 4:** Linear regressions of agreement with the content of the petition onto the SEIS scale, controlling for relevant covariates.

	Model 1: SEIS only		Model 2: SEIS plus covariates	
	*b*	SE	*b*	SE
Intercept	7.29***	0.25	5.83***	0.86
SEIS	-0.68***	0.07	-0.61***	0.09
Age	—	—	-0.01	0.01
Gender	—	—	0.38	0.23
Education	—	—	0.42*	0.18
Household Income	—	—	0.02	0.06
Political Ideology	—	—	-0.08	0.08
Childhood Inequality	—	—	0.06	0.08
**Adjusted R**^**2**^	.41		.45	

*Note*.

* p < .05

** *p* < 0.01

*** *p* < 0.001

**Table 5:** Logistic regressions of whether or not the petition was signed onto the SEIS scale, controlling for relevant covariates.

	Model 1: SEIS only		Model 2: SEIS plus covariates	
	*b*	SE	*b*	SE
Intercept	0.91***	0.41	-5.26**	1.82
SEIS	-0.45**	0.15	-0.51*	0.22
Age	—	—	-0.01	0.02
Gender	—	—	0.99*	0.45
Education	—	—	0.88*	0.37
Household Income	—	—	0.19	0.13
Political Ideology	—	—	-0.04	0.17
Childhood Inequality	—	—	0.48**	0.18

*Note*. For petition signing, 0 = did not sign, 1 = did sign.

* p < .05

** *p* < 0.01

*** *p* < 0.001

### Discussion

Study 4 provides initial evidence for the predictive validity of the SEIS. Individuals reporting greater support for economic inequality are less likely to agree with and sign a petition to increase the minimum wage, even when controlling for age, gender, education, household income, political ideology, and level of inequality in a participant’s childhood neighborhood. Notably, support for economic inequality was the strongest predictor of expressed petition agreement and signing behavior in the present data. These findings demonstrate the predictive validity of the SEIS, indicating that it captures feelings toward economic inequality and predicts willingness to act against it.

However, Study 4 had three limitations. First, Study 4 had a relatively small sample size. In Study 5 we recruited a larger sample. Second, Study 4 did not compare the predictive validity of the SEIS to existing measures which are conceptually similar, such as economic system justification [18] or belief in a just world [48]. These measures capture related constructs but are more conceptually broad. As such, the SEIS, which aims to solely measure support for economic inequality, should offer a more precise assessment of this construct. In Study 5, we tested whether this is the case. Finally, in Study 4 we measured agreement and behavior before participants completed the SEIS, meaning responses on the SEIS could have been biased by recent action. Therefore, in Study 5, participants completed SEIS before being provided with an opportunity to enact support for (in)equality.

## Study 5

The most important extension that we offer in Study 5 is the demonstration that the SEIS is conceptually distinct, and thus measures something different, from currently existing measures of Social Dominance Orientation, Belief in a Just World, Economic System Justification, Protestant Work Ethic, and Inegalitarianism. Importantly, past research has demonstrated that comparing different measures with a multiple regression can result in inflated Type I errors [56]. Therefore, we followed the structural equation modeling procedures laid out by Westfall and Yarkoni [56] in order to explore whether the SEIS is separable from these related psychological constructs. If the SEIS is indeed distinct from each of the conceptually related measures listed above, we would expect that the SEIS explains more of the latent variance in donation amount relative to the five other predictors [57].

### Participants

We collected data from 652 participants (M_age_ = 39.50, 52.9% Female, Political Party identification: 41.3% Democrat, 26.6% Independent, 25.3% Republican, 3.7% Other, *Median*_*income*_ *=* $50,000 - $59,999) on mTurk.

### Procedure

Participants were first presented with a survey containing all of our potential predictor variables, in random order: the SEIS, perceptions of the level of inequality, perceptions of growth in inequality, belief that inequality is unfixable, belief in a just world [48], social dominance orientation [58], economic system justification [18], protestant work ethic [59], inegalitarianism [19], support for redistribution [20], and the single-item measures of support for inequality from the World Values Survey [20] and International Social Survey Programme [60].

Following this, participants were presented with an opportunity to make a proxy-donation to an organization called “Fight for $15.” Specifically, participants were told that they would be given ten raffle tickets for a draw to win one of four $25 bonus payments. Participants then read a short paragraph which described Fight for $15 as “an advocacy organization that is fighting to reduce economic inequality by raising the minimum wage … from $7.25 to $15 an hour nationwide” (See https://osf.io/cmzye/ for full text). After, participants were asked to decide “how many of [their] 10 raffle tickets [they would] like to transfer to the Fight for $15.” Lastly, participants reported their demographics.

### Results

#### Correlations

We first ran a series of correlations between the SEIS and all the predictor variables. Unsurprisingly, the SEIS was strongly correlated with conceptually similar measures, including social dominance orientation (r = .77, *p* < .001), economic system justification (r = .78, *p <* .001), and support for redistribution (r = .75, *p* < .001; Table 6). While these correlations appear high, it is worth pointing out that they are no larger than the correlations between several of the other related constructs. For example, in our sample social dominance orientation with economic system justification (r = .75, *p* < .001) or inegalitarianism (r = .68, *p* < .001), inegalitarianism with economic system justification (r = .78, *p* < .001), as well as the WVS measure of support for economic inequality and economic system justification (r = .67, *p* < .001).

**Table 6:** Correlations between all predictor variables in Study 5.

	*1*.	*2*.	*3*.	*4*.	*5*.	*6*.	*7*.	*8*.	*9*.	*10*.	*11*.	*12*.
1. SEIS	—											
2. PI	-.55	—										
3. GI	-.56	.40	—									
4. IU	-.68	.49	.44	—								
5. BJW	.27	-.46	-.26	-.29	—							
6. SDO	.77	-.55	-.42	-.65	.22	—						
7. ESJ	.78	-.56	-.49	-.77	.38	.75	—					
8. PWE	.40	-.46	-.29	-.47	.47	.39	.54	—				
9. EGAL	.71	-.55	-.48	-.72	.37	.68	.78	.59	—			
10.SR	-.75	.38	.44	.59	-.11	-.63	-.68	-.28	-.56	—		
11. WVS	.72	-.58	.43	-.64	.31	.69	.67	.43	.66	-.57	—	
12. ISSP	-.75	.45	.49	.50	-.19	-.62	-.66	-.26	-.55	.65	-.62	—

*Note*. SEIS = Support for Economic Inequality; PI = Perceived Inequality; GI = Perceived Growth in Inequality, IU = Belief that Inequality is Unfixable; BJW = Belief in a Just World; SDO = Social Dominance Orientation; ESJT = Economic System Justification; PWE = Protestant Work Ethic; EGAL = Inegalitarianism; SR = Support for Redistribution; WVS = World Values Survey measure of Support for Inequality; ISSP = International Social Survey Programme measure of Support for Inequality. all p values < .001.

While the correlations between the SEIS and other conceptually similar measures may appear high, these correlations are no higher than what is common between previously published measures that are considered conceptually distinct (e.g., SDO and Right Wing Authoritarianism, r = .60, [61]). As underlined earlier, the SEIS is meant to measure a construct that could be considered a subset of many of the above-mentioned measures and thus we expect relatively high correlations. For example, whereas SDO assesses preferences for group-based dominance hierarchies, which can include economic stratification, SEIS measures tolerance for social hierarchies specifically rooted in economic inequality. In order to ensure that the SEIS is conceptually distinct from each of these measures, and thus offers unique information in predicting various behaviours, we followed up these correlations with assessments of incremental validity and separability of constructs

#### Incremental validity

To first explore how the SEIS performs relative to the previously established measures we ran one large regression containing all relevant predictors (Table 6). In this model, only the SEIS, economic system justification, the WVS measure of support for economic inequality, and support for redistribution significantly predicted how much someone chose to donate to the Fight for $15. The only measure which predicted donations stronger than the SEIS was the WVS measure of support for economic inequality. However, this particular finding highlights the challenges with multicollinearity: the WVS measure of support for economic inequality predicts donations to the Fight for $15 in the opposite direction in which we would predict. That is, stronger endorsement of the statement “we need larger income differences as incentives” actually predicts more donations to the Fight for $15. High multicollinearity often results in large standard errors, and thus unstable estimates of regression coefficients [62].

**Table 7:** Single regression model containing all predictors in Study 5 on raffle ticket donations.

	Base Model	
	*β*	SE
Intercept	.00	.04
SEIS	-.16*	.08
Belief in a Just World	.04	.04
Social Dominance Orientation	.05	.07
Economic System Justification	-.16*	.08
Protestant Work Ethic	.09	.05
World Values Survey	.21***	.06
International Social Survey Programme	.07	.06
Inegalitarianism	-.01	.07
Support for Redistribution	.17**	.06

*Note*.

* p < .05

** *p* < 0.01

*** *p* < 0.001

Given the high degree of correlation between all the predictors, we computed the Variance Inflation Factors (VIF) for each predictor as well as the Klein test for multicollinearity [59]. We found that the VIFs were all generally quite large (e.g., > 1.5 average 2.94), and that Klein’s test detected multicollinearity issues with every single predictor (S7 Table). As mentioned previously, past work has demonstrated that attempting to control for other variables using linear regressions can result in inflated Type I errors [56]. Thus, in order to accurately assess whether the SEIS is distinct from the conceptually related constructs, we utilized a structural equation modeling approach [57].

#### Separability of constructs

We built a model where latent support for economic inequality, belief in a just world, social dominance orientation, economic system justification, protestant work ethic, and inegalitarianism predicted how many raffle tickets were donated to the Fight for $15. All latent construct covariances were free to vary, however we present the model with these paths omitted simply for clarity (S8 Table).

**Fig 4:**
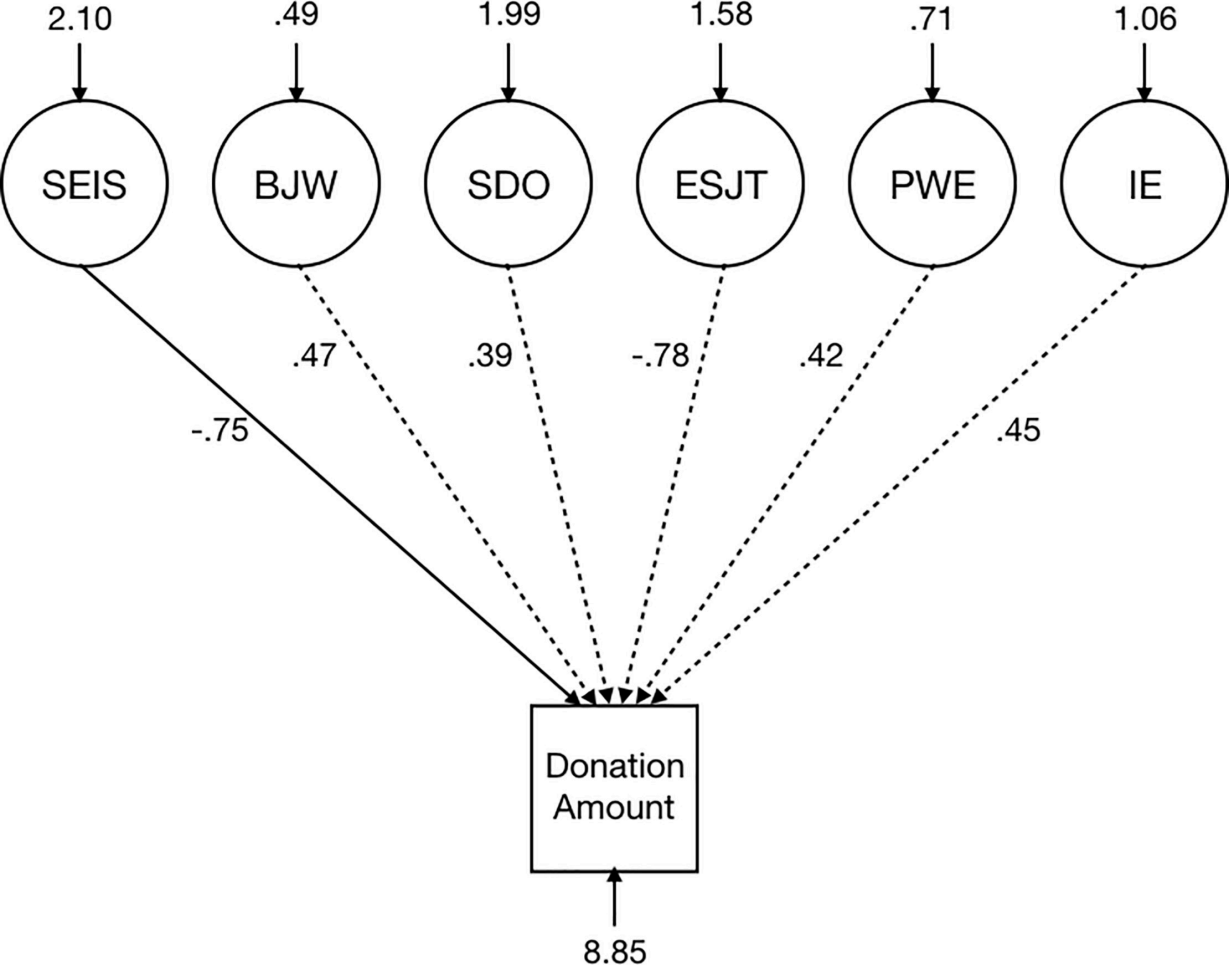
Analysis of separability of constructs in Study 5. A structural equation model demonstrating that the SEIS was the only significant predictor of donation amount across six conceptually similar measures.

If the SEIS is conceptually distinct, we would expect that it would explain more of the latent variance in donation amount than the other included latent constructs. Testing this model supported our hypothesis that support for economic inequality is distinct from these conceptually similar constructs. Latent support for economic inequality was the only significant predictor of donation amount after controlling for all other variables, with the standardized coefficient for latent support for economic inequality = -.75, *p* = .004 (Fig 4). These results suggest that, while correlated, the SEIS is distinct from other conceptually similar measures.

## Discussion

Study 5 offers additional evidence for the predictive validity of the SEIS by showing that it can predict donations to an organization fighting for minimum wage better other conceptually similar measures. Additionally, the SEIS showed similar or superior predictive validity to single item measures of support for economic inequality (i.e., the WVS and ISSP measures), but does not have the same drawbacks as a single-item measure, such as the inability to assess reliability and properly evaluate its psychometric properties. Most crucially, in Study 5 we demonstrated with a structural equation model that support for economic inequality is indeed conceptually distinct from other conceptually similar measures. In this analysis we found that the SEIS explained some of the latent variance in donation amount (i.e., people who demonstrated more support for inequality donated less to helping increase the minimum wage) above and beyond related measures. This finding supports our claim that while measures like economic system justification and social dominance orientation may capture aspects of support for inequality, there is a benefit to utilizing our measure, which more directly assesses support for economic inequality, when exploring attitudes towards economic inequality. That is, the SEIS can be used when one wishes to more directly assess attitudes towards economic inequality without also invoking elements of group dominance (i.e., social dominance), hard work (e.g., Protestant Work Ethic), or the entire economic system (e.g., Economic System Justification). Given the recent rise in research focusing specifically on attitudes towards economic inequality (e.g., [63–65]), such a tool should prove a useful.

## Differential item functioning

One final element to assessing the psychometric fitness of the SEIS is exploring whether the scale’s items function similarly in different populations. As such, we conducted a set of Differential Item Functioning (DIF) analyses across both (a) political ideology and (b) relative high and low income.

To achieve the recommended minimum sample size of 300 per subgroup [66], we combined the data from Studies 2 and 5 (n_total_ = 1,308). We used these two studies because they were the only studies where participants identified themselves as either a Republican or Democrat and completed the worldwide version of the SEIS.

For DIF analyses we explored how the items functioned (ICCs, discrimination, information, and unidimensionality) among both Democrats and Republicans (n_dem_ = 535, n_rep_ = 343). Second, we conducted a median split on income and explored how the items functioned in the (relative) high and low-income categories. The median income was $40,000-$49,999, thus the low-income category contains participants with household incomes under $50,000 (n_low_ = 658) and the high-income category contains participants with a household income of $50,000 and above (n_high_ = 649). Discrimination parameters and Information values can be found in S9–S12 Tables; ICCs can be found at https://osf.io/cmzye/.

### Item functioning by political party identification

The SEIS items functioned similarly across Republicans and Democrats, with some minor differences. Specifically, each of the five items demonstrated sharp and clearly defined ICCs across both groups, indicating that the five items function with a high degree of discrimination and are effective and differentiating between people in their underlying support for economic inequality. Interestingly, for Democrats, the five items appear to better differentiate among those high on latent support for inequality. This is possibly because self-identified Democrats express lower support for economic inequality overall. Thus, they must possess a reasonably high degree of the underlying trait to select more extreme Likert scale responses. For Republicans, however, the ICCs are much more centered along the x-axis. This suggests that the SEIS is better at differentiating between Republicans along the spectrum of latent support for economic inequality, as opposed to simply on the high end.

Across both groups, discrimination values were slightly lower than those observed in Studies 1–3, however values were all greater than 1.5 (with most still above 3), suggesting the items adequately differentiate between people on underlying support for economic inequality regardless of political affiliation [36]. Additionally, in both Democrats and Republicans, all items contributed a high degree of information to the total scale (Information_dem_ = 57.41 and Information_rep_ = 50.38). Consistent with Studies 1–3, Items 3 and 10 appear to be the weakest items in both the Republican and Democrat subgroups. Additionally, these two items appear to function better in Democrats than Republicans–this can be seen in both the flatter ICCs, lower discrimination, and lower Information values present in Republicans. Notably, the pattern of discrimination parameters and information values are roughly equivalent to the patterns seen in Studies 1–3. For example, among Republicans and Democrats items 3,10 and 18 contribute a similar proportion of the scale’s total information (S13 Table).

While two items (i.e., “The negative consequences of economic inequality have been largely exaggerated” and “Economic inequality is not a problem”) appear to be less discriminating among Republicans, they still pass the discrimination thresholds for being informative items. Moreover, in both Republicans and Democrats, the five-item SEIS passes our threshold for unidimensionality with the chi-square values for each item not exceeding three times the corresponding degrees of freedom (S13 Table; [46]). Thus, the SEIS appears unidimensional regardless of respondent’s political affiliation.

### Item functioning by income

Across both the low- and high-income groups, each of the five items demonstrated sharp and clearly defined ICCs, indicating a high degree of discrimination based on underlying support for economic inequality. Interestingly, the ICCs show that the SEIS appears to be better at differentiating between low-income people who are higher in latent support for economic inequality, and high-income people lower in latent support for economic inequality. Across both the low- and high-income groups, all discrimination values were above 2 (with most still above 3), suggesting the items adequately differentiate between people regardless of their relative low- or high-income status [37]. Additionally, in both low- and high-income participants, all items contributed a high degree of information to the total scale (Information_LI_ = 58.12 and Information_HI_ = 61.04). Consistent with Studies 1–3, items 3 and 10 appear to be slightly weaker items in both the low- and high-income subgroups. However, these two items appear to function equivalently across the two income subgroups.

Thus, the SEIS functions similarly in both the low- and high-income subgroups, compared with the overall individual item analyses from Studies 1–3. Moreover, in both low- and high-income participants, the five-item SEIS passes our threshold for unidimensionality with the chi-square values for each item not exceeding three times the corresponding degrees of freedom (S15 Table), and it appears that the SEIS is unidimensional regardless of the respondent’s income.

## General discussion

As economic inequality has risen, so, too, has its status as a topic of interest to researchers, politicians, policy makers, and the public. However, there are presently no psychometrically adjudicated and validated measures of support for economic inequality. This paper seeks to fill the gap by providing a psychometrically sound support for economic inequality scale. Across five studies we employ Item Response Theory [33] to construct, evaluate, and validate a five-item scale measuring support for economic inequality in two different framing contexts: the world and the United States. The scale demonstrates favorable psychometric properties (individual item functioning and unidimensionality), high reliability, predictive validity, as well as convergent and discriminant validity in both the worldwide and United States contexts.

Using this measure, future researchers have an efficient and effective tool for measuring support for economic inequality. As mentioned previously, most research studying related constructs (e.g., general perceptions of inequality) have relied on either face valid measures. While these data are valuable in uncovering initial relationships, we hope the present measure aids researchers in understanding how people develop support for economic inequality, as well as the consequences of this attitude. For example, the SEIS could be used to assess the effectiveness of interventions designed to reduce support for economic inequality or understand the causes of support for economic inequality [67].

### Limitations and future directions

The current research provides a new and effective measure of support for economic inequality, but there are numerous aspects of the scale still to be explored. One potential limitation is the overall skewness of the responses to the scale. However, non-normality is common in psychology [40], and the observed positive skew may indicate that most people have low levels of support for economic inequality as seen in our samples. Consistent with this explanation, in a large cross-national sample of Europeans (n = 54,059) the mean response to the question “Income differentials in [my] country are too large” is 4.23 on a 5-point Likert scale, where 5 is strongly agree (skewness = -1.34, kurtosis = 1.70; [60]). This suggests that many people are strongly intolerant of inequality. As such, we do not interpret the skewness in the data to be problematic, but rather a reflection of how this construct may exist in the population.

One additional concern regarding the SEIS is a potential disconnect between explicitly reported attitudes and behaviors surrounding economic inequality. For instance, it is possible that someone may report a low degree of support for economic inequality but engage in behaviour that supports it. One example of this phenomenon is the tendency for people who live in poverty (i.e., a population that should not support the current level of economic inequality) to vote for policies that go against their own economic self-interests and help maintain the status-quo [68]. Attitudes often do not always predict behavior [69], but this does not mean measuring attitudes is a worthless endeavor. Whether or not the attitude of support for economic inequality predicts relevant behavior is an empirical question examined here. Two findings from the current work suggest this potential disconnect between attitudes and behaviour is of minimal concern for the present measure. First, in Studies 4 and 5, scores on the SEIS significantly predict behaviour aimed at mitigating economic inequality via helping the poor. This suggests that the SEIS at least moderately correlates with relevant behaviors. Secondly, in Study 3 we did not find any relationship between the SEIS and the measures of social desirability and self-enhancement. This suggests that people who might be supportive of economic inequality are not simply attempting to make themselves look socially desirable by reporting low support for economic inequality. Future research may further explore this possibility of a disconnect by testing how the SEIS relates to specific economic policy preferences or real-world behaviours such as voting.

One strength of the present scale is its flexibility. Specifically, researchers can modify the measure to different levels and contexts with ease. For example, the SEIS can be adapted to assess support for economic inequality in any other country, a specific state, city, county, community, etc. We explored one of these iterations–the United States–and encourage researchers to use the scale in the context appropriate for their research question, with proper adjudication of the altered scale. Researchers should not assume the items will function as they did here when content has been changed, though the results of Study 3 suggests that the scale can be easily adapted to different populations.

Using the SEIS, researchers can explore the consequences of support for economic inequality and build an understanding of the social and psychological factors influencing the development of (non)support for economic inequality. Moreover, researchers interested in support for economic inequality can use the SEIS to explore how it relates to non-self-report responses to economic inequality (e.g., physiological arousal). Finally, as researchers use the SEIS across different contexts, we stand to further strengthen evidence for its psychometric fitness, reliability, and validity.

## Supporting information

S1 Fig Study 1 Item Characteristic Curve for Item 1. (PNG)

S2 Fig Study 1 Item Characteristic Curve for Item 2. (PNG)

S3 Fig Study 1 Item Characteristic Curve for Item 3. (PNG)

S4 Fig Study 1 Item Characteristic Curve for Item 4. (PNG)

S5 Fig Study 1 Item Characteristic Curve for Item 5. (PNG)

S6 Fig Study 1 Item Characteristic Curve for Item 6. (PNG)

S7 Fig Study 1 Item Characteristic Curve for Item 7. (PNG)

S8 Fig Study 1 Item Characteristic Curve for Item 8. (PNG)

S9 Fig Study 1 Item Characteristic Curve for Item 9. (PNG)

S10 Fig Study 1 Item Characteristic Curve for Item 10. (PNG)

S11 Fig Study 1 Item Characteristic Curve for Item 11. (PNG)

S12 Fig Study 1 Item Characteristic Curve for Item 12. (PNG)

S13 Fig Study 1 Item Characteristic Curve for Item 13. (PNG)

S14 Fig Study 1 Item Characteristic Curve for Item 14. (PNG)

S15 Fig Study 1 Item Characteristic Curve for Item 15. (PNG)

S16 Fig Study 1 Item Characteristic Curve for Item 16. (PNG)

S17 Fig Study 1 Item Characteristic Curve for Item 17. (PNG)

S18 Fig Study 1 Item Characteristic Curve for Item 18. (PNG)

S19 Fig Study 2 Information function for the three candidate compositing rules. *Note*. The solid line is the Maximum Likelihood estimated theoretical maximum information, the dashed line is the *a*_*j*_ weighted composite information, and the dotted line is the unit-weighted composite information.
(PNG)

S20 Fig Study 2 Item Characteristic Curve for Item 1. (PNG)

S21 Fig Study 2 Item Characteristic Curve for Item 2. (PNG)

S22 Fig Study 2 Item Characteristic Curve for Item 3. (PNG)

S23 Fig Study 2 Item Characteristic Curve for Item 4. (PNG)

S24 Fig Study 2 Item Characteristic Curve for Item 5. (PNG)

S25 Fig Study 3 Information function for the three candidate compositing rules. *Note*. The solid line is the Maximum Likelihood estimated theoretical maximum information, the dashed line is the *a*_*j*_ weighted composite information, and the dotted line is the unit-weighted composite information.
(PNG)

S1 Table Graded model parameter estimates for the full 18 items in Study 1. *Note*. Standard Errors for each parameter are in brackets, *a* is the item’s discrimination parameter, *bs* are the thresholds.
(DOCX)

S2 Table Graded model item parameter estimates for the Final 5 Items in Study 1. *Note*. Standard Errors for each parameter are in brackets. a is the item’s discrimination parameter, b are the thresholds.
(DOCX)

S3 Table Item and total scale information for the Final 5 Items in Study 1. (DOCX)

S4 Table Goodness-of-fit Chi-Square tests for the 5 item scale in Study 2. (DOCX)

S5 Table Goodness-of-fit Chi-Square tests for the five item scale in Study 3. (DOCX)

S6 Table Correlations between all scales assessing convergent validity in Study 3. (DOCX)

S7 Table Assessment of multicollinearity in Study 5. (DOCX)

S8 Table Full SEM model output in Study 5. (DOCX)

S9 Table Graded model item parameter estimates in low household income. *Note*. Standard Errors for each parameter are in brackets. a is the item’s discrimination parameter, b are the thresholds.
(DOCX)

S10 Table Item and total scale information for low household income. (DOCX)

S11 Table Graded model item parameter estimates in high household income. *Note*. Standard Errors for each parameter are in brackets. a is the item’s discrimination parameter, b are the thresholds.
(DOCX)

S12 Table Item and total scale information for high household income. (DOCX)

S13 Table Item and total scale information for Democrats and Republicans. (DOCX)

S14 Table Goodness-of-fit Chi-Square tests for the five-item scale for Democrats and Republicans. (DOCX)

S15 Table Goodness-of-fit Chi-Square tests for the five-item scale in low and high household income. (DOCX)
