# Supplementary figures and images for: The Support for Economic Inequality Scale: Development and adjudication
Source: PLoS One. 2019 Jun 21;14(6):e0218685. doi: 10.1371/journal.pone.0218685 (PMC6588246; doi:10.1371/journal.pone.0218685)

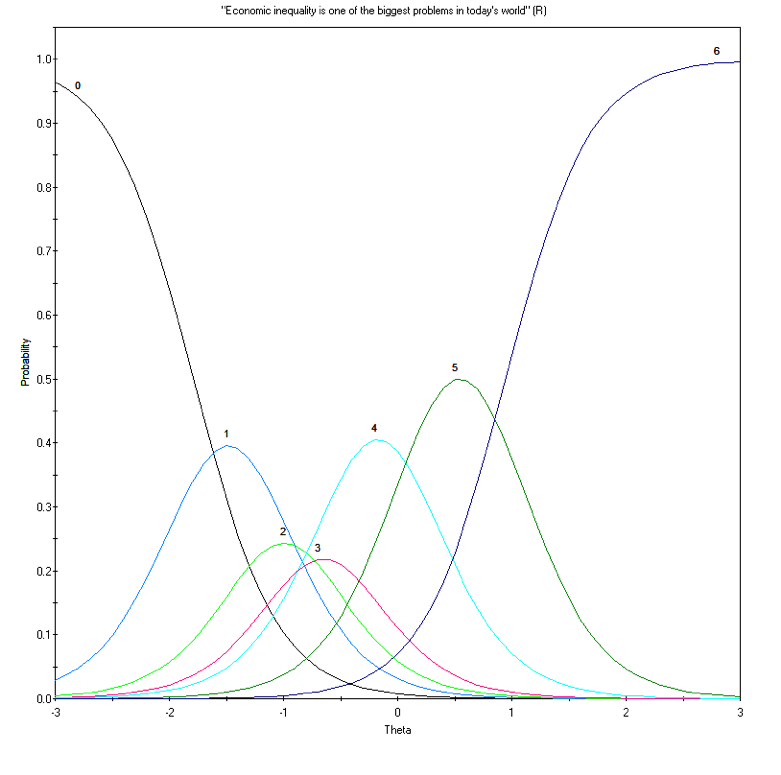

Supplement: S1 Fig — (PNG) [file pone.0218685.s001.png]

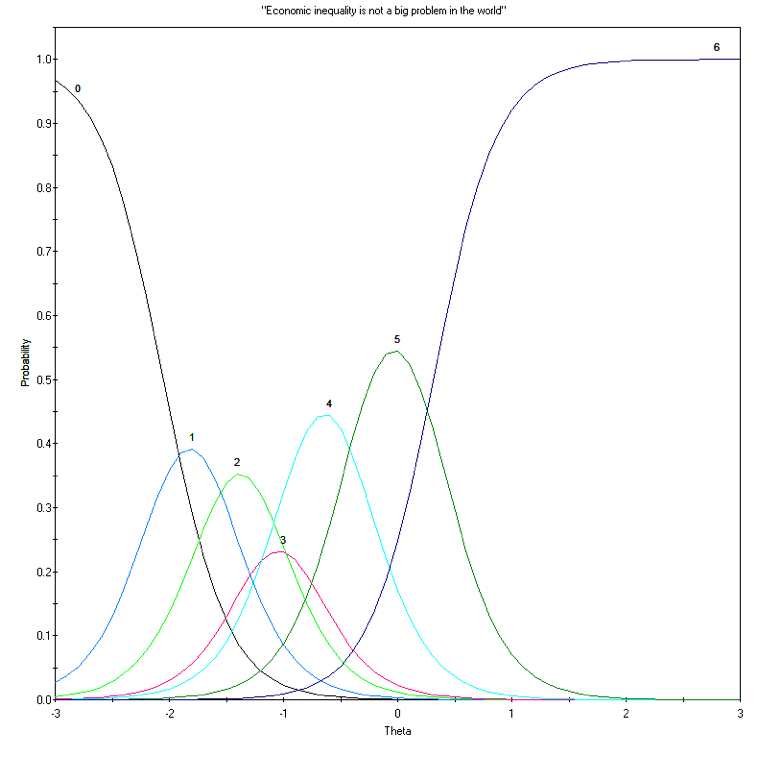

Supplement: S2 Fig — (PNG) [file pone.0218685.s002.png]

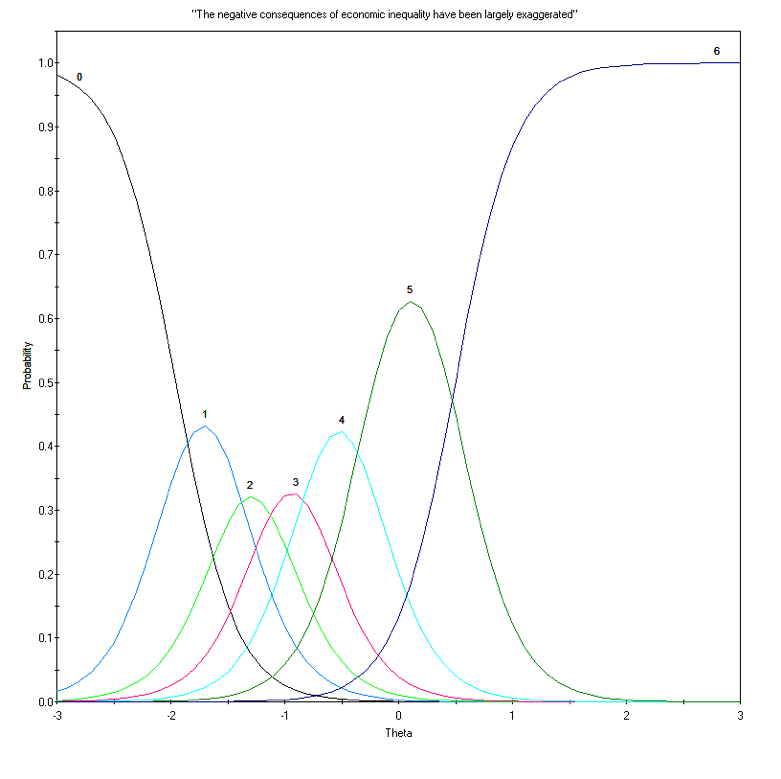

Supplement: S3 Fig — (PNG) [file pone.0218685.s003.png]

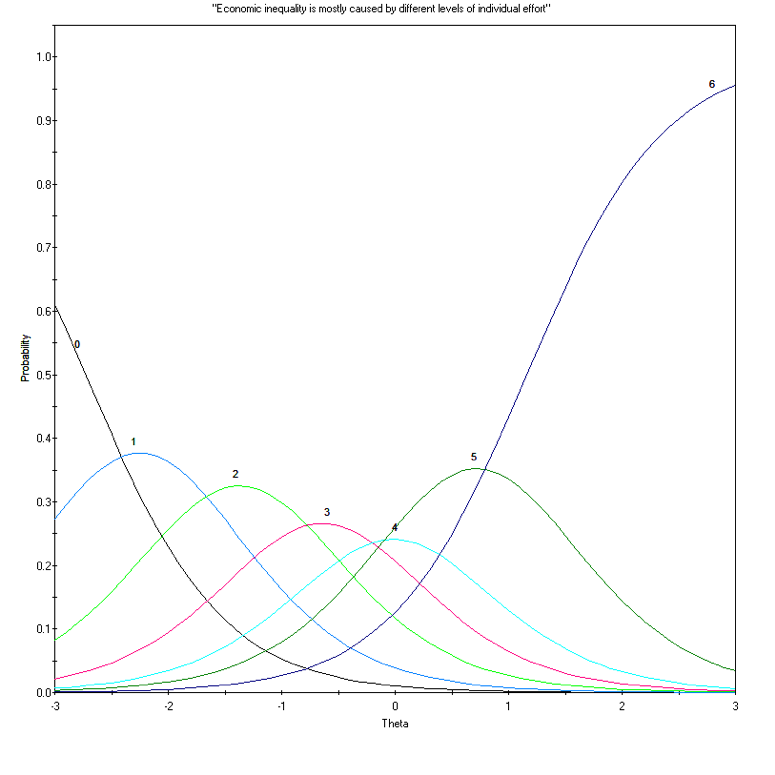

Supplement: S4 Fig — (PNG) [file pone.0218685.s004.png]

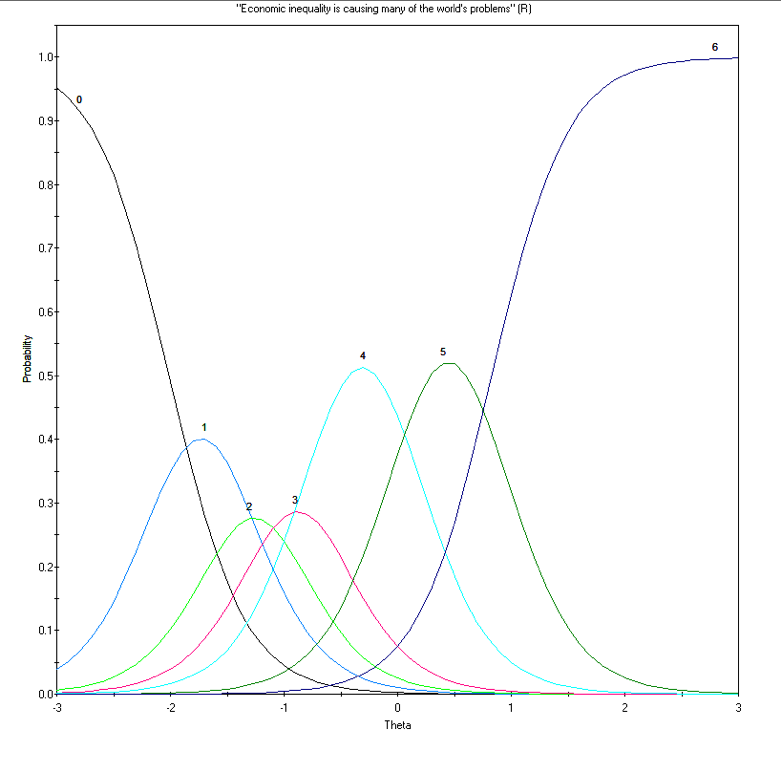

Supplement: S5 Fig — (PNG) [file pone.0218685.s005.png]

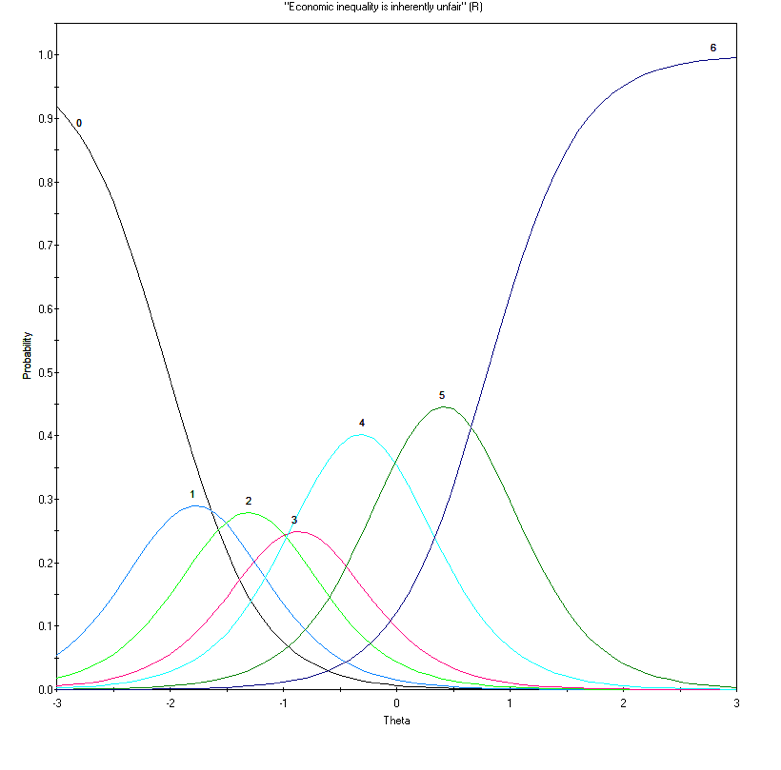

Supplement: S6 Fig — (PNG) [file pone.0218685.s006.png]

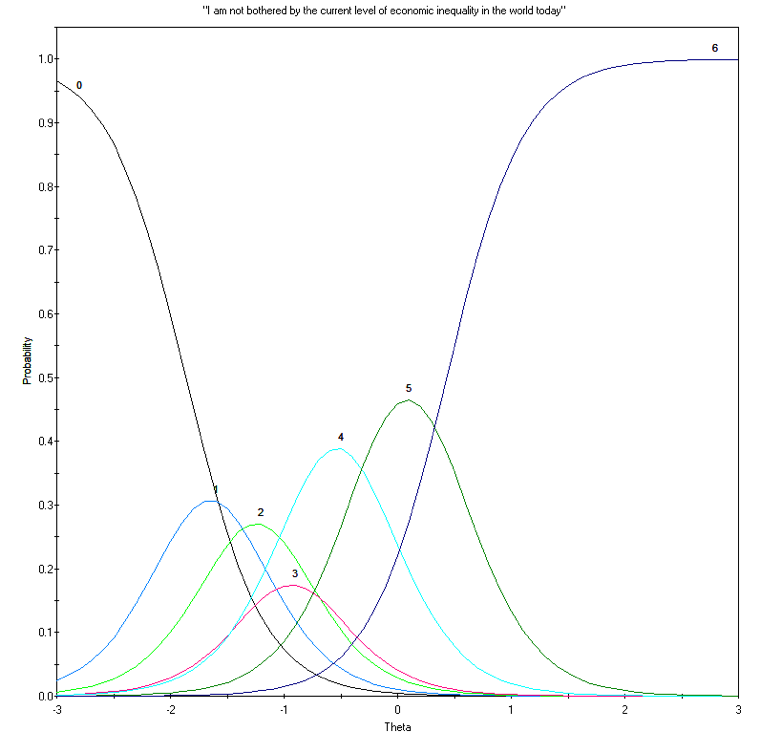

Supplement: S7 Fig — (PNG) [file pone.0218685.s007.png]

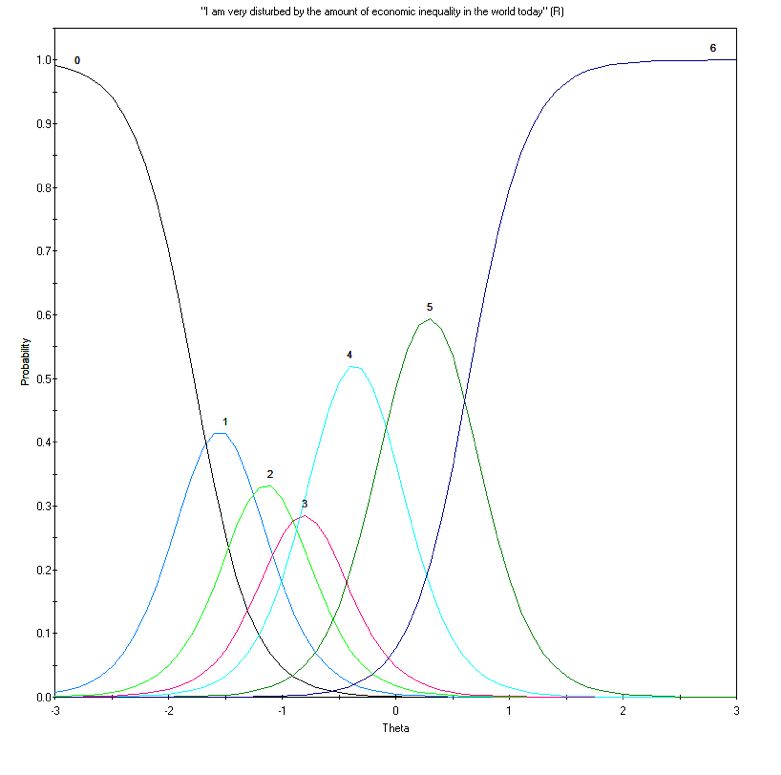

Supplement: S8 Fig — (PNG) [file pone.0218685.s008.png]

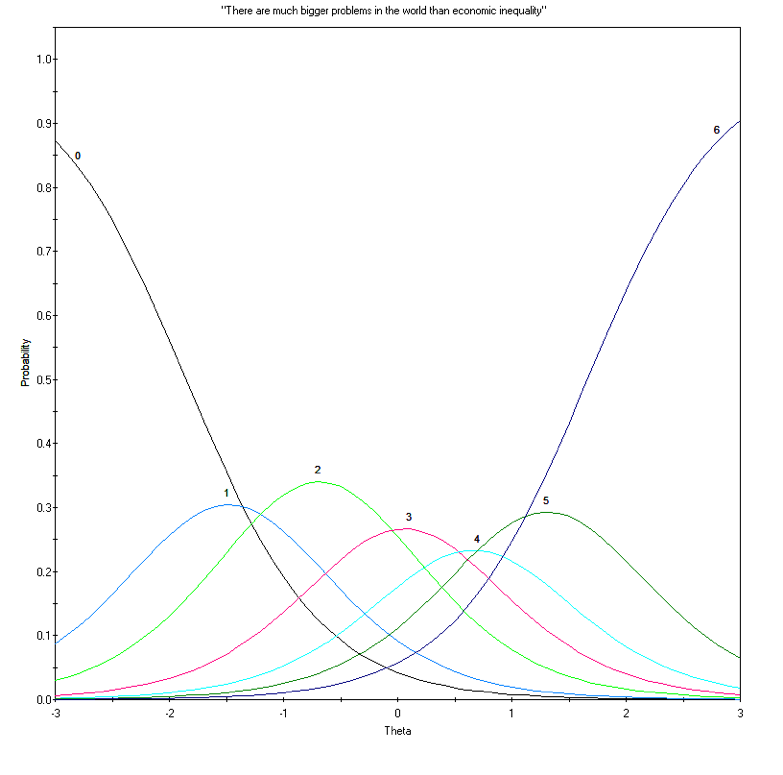

Supplement: S9 Fig — (PNG) [file pone.0218685.s009.png]

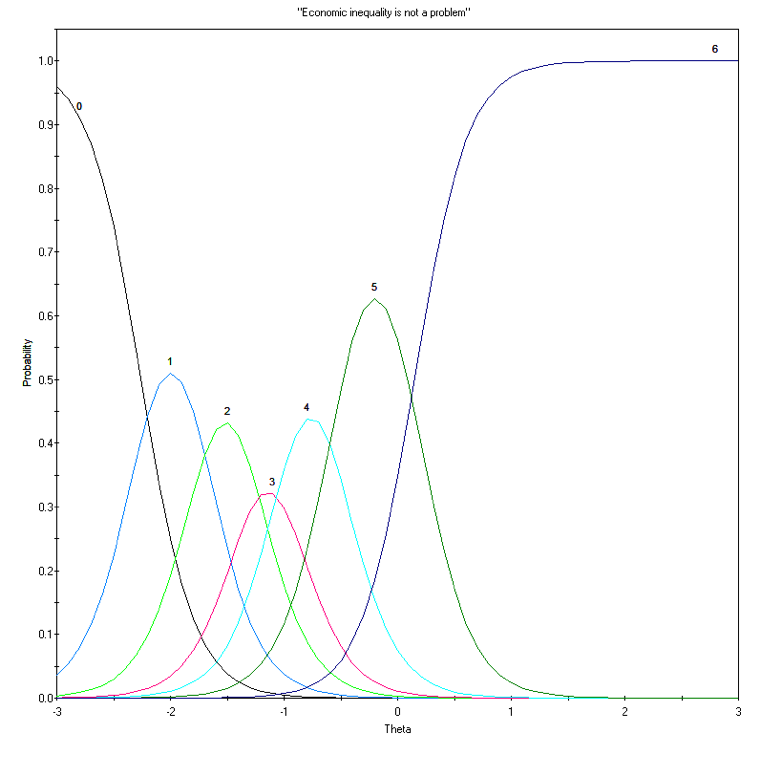

Supplement: S10 Fig — (PNG) [file pone.0218685.s010.png]

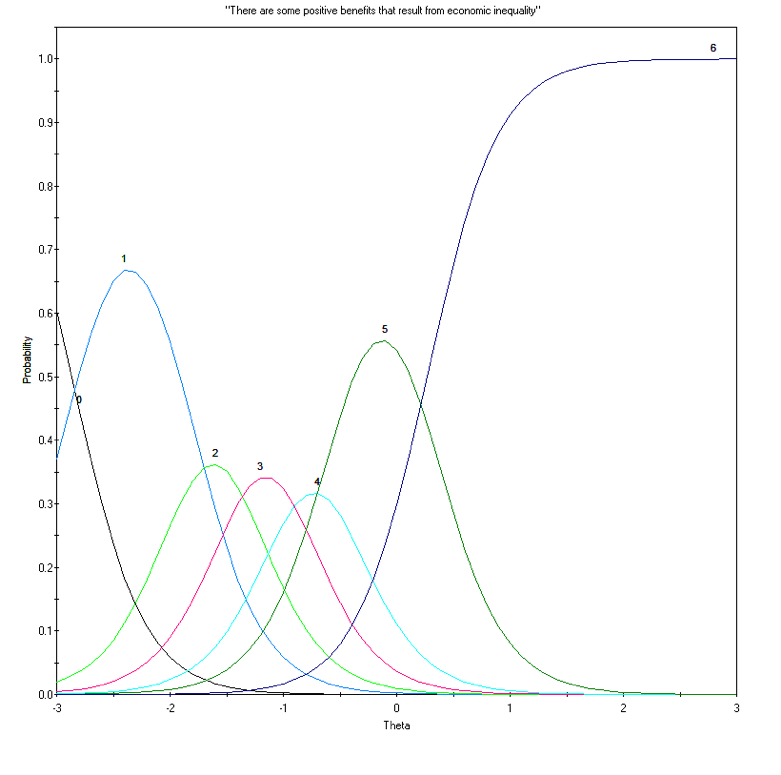

Supplement: S11 Fig — (PNG) [file pone.0218685.s011.png]

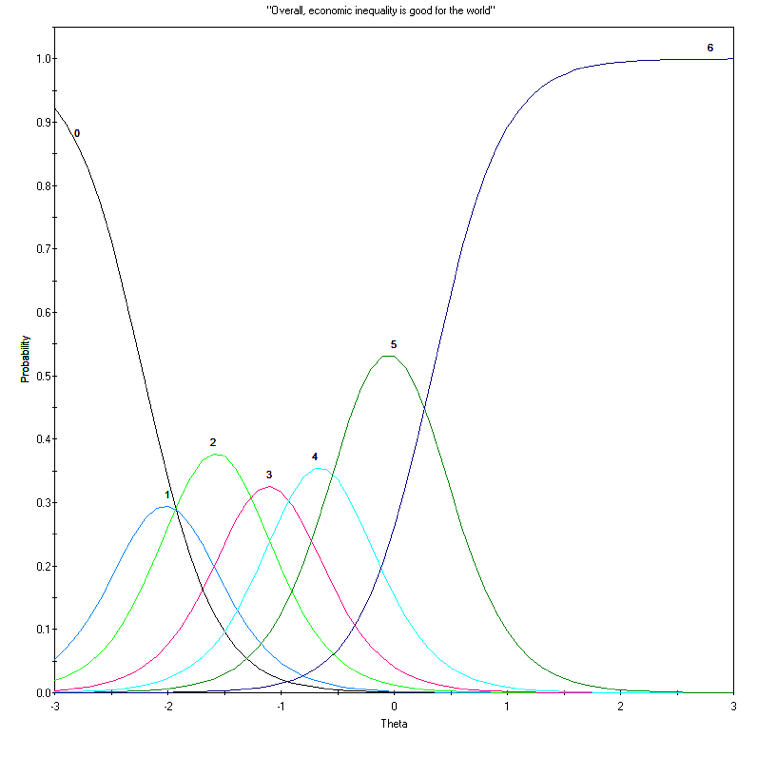

Supplement: S12 Fig — (PNG) [file pone.0218685.s012.png]

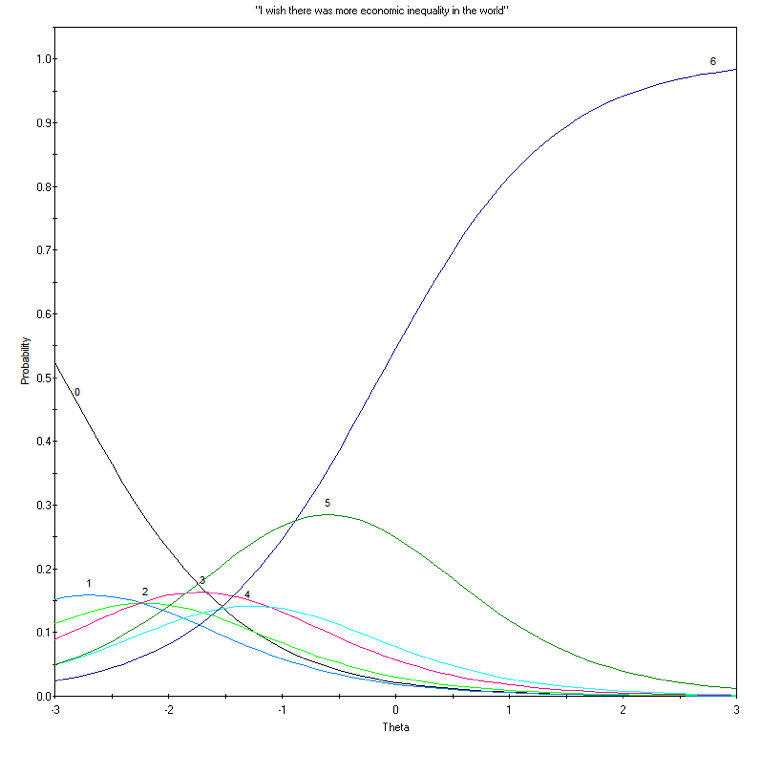

Supplement: S13 Fig — (PNG) [file pone.0218685.s013.png]

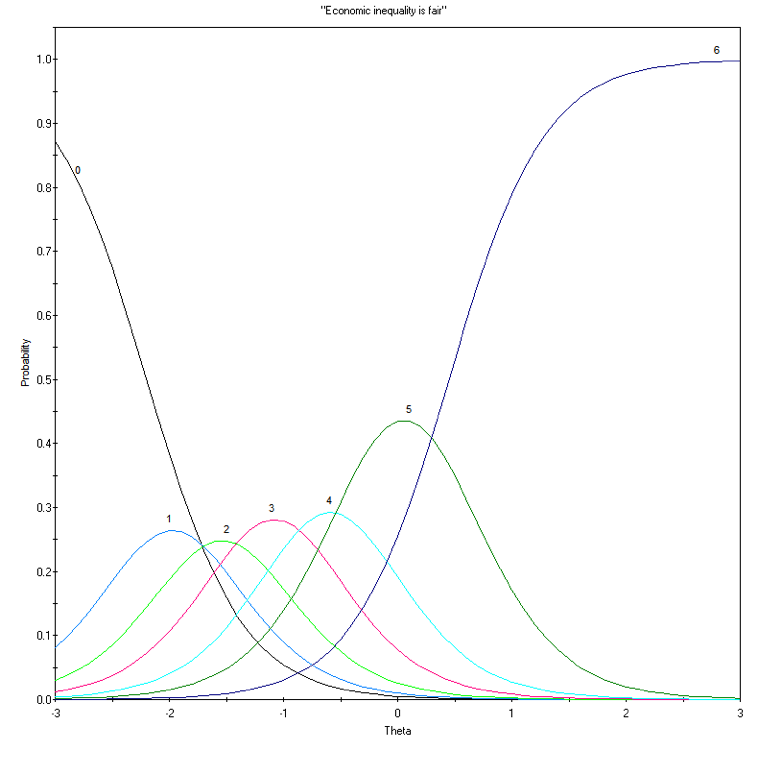

Supplement: S14 Fig — (PNG) [file pone.0218685.s014.png]

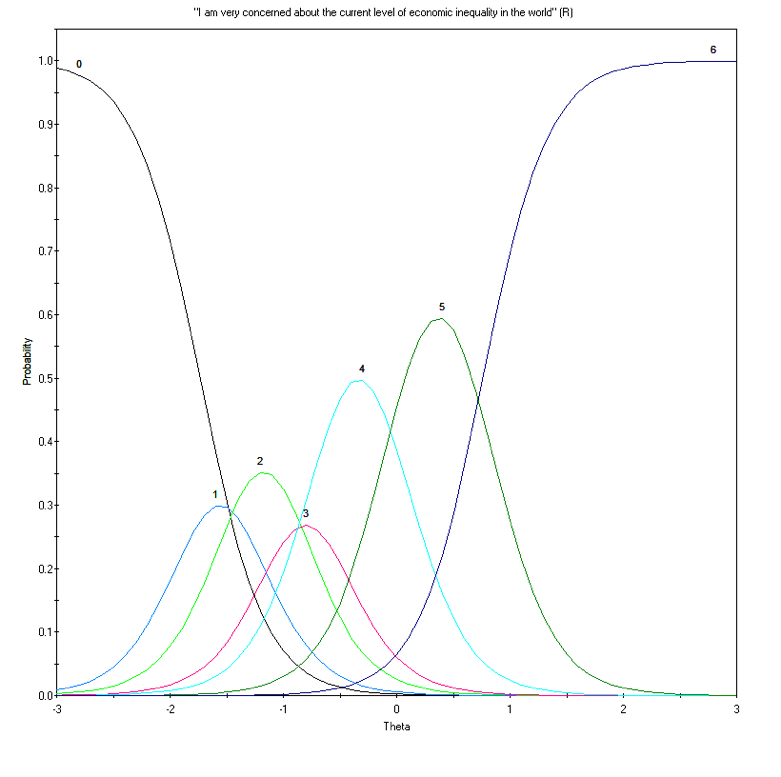

Supplement: S15 Fig — (PNG) [file pone.0218685.s015.png]

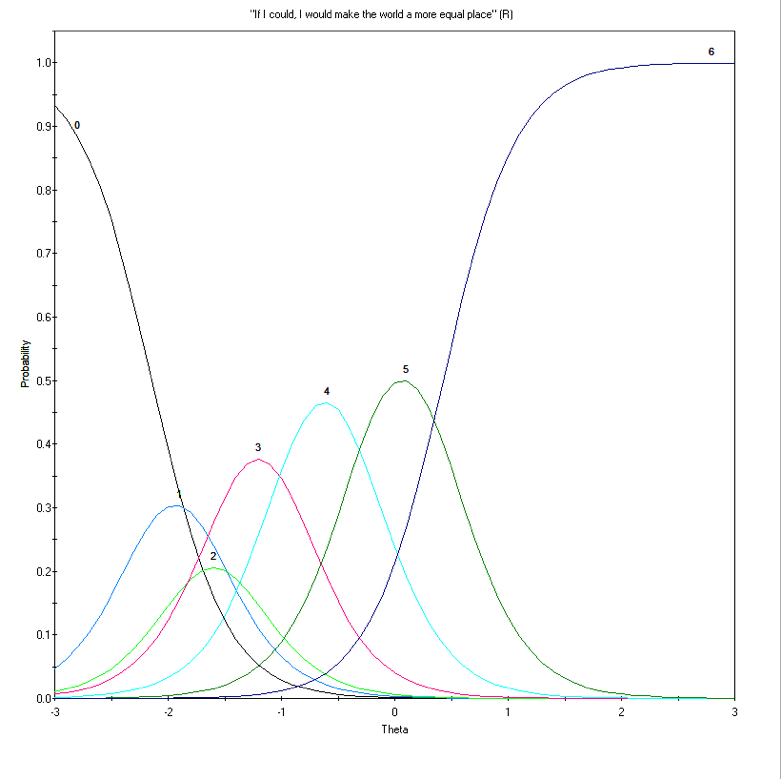

Supplement: S16 Fig — (PNG) [file pone.0218685.s016.png]

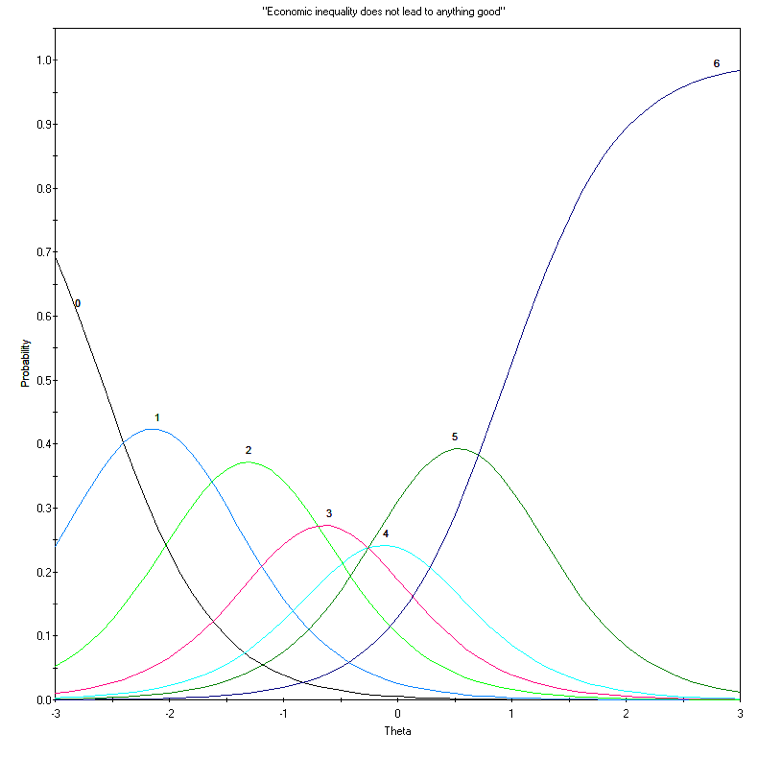

Supplement: S17 Fig — (PNG) [file pone.0218685.s017.png]

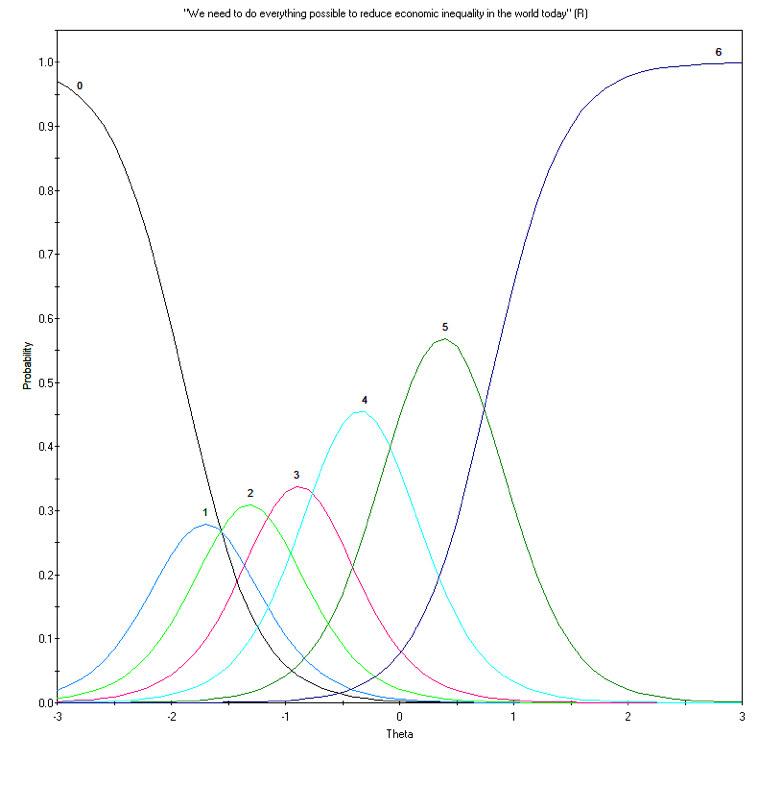

Supplement: S18 Fig — (PNG) [file pone.0218685.s018.png]

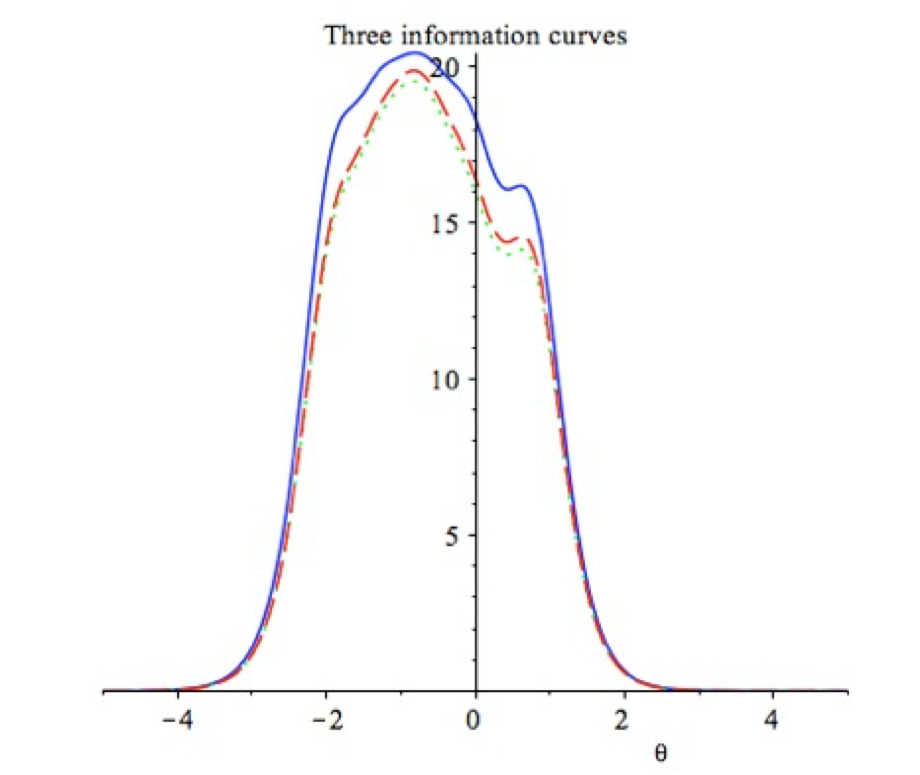

Supplement: S19 Fig — Note. The solid line is the Maximum Likelihood estimated theoretical maximum information, the dashed line is the aj weighted composite information, and the dotted line is the unit-weighted composite information. (PNG) [file pone.0218685.s019.png]

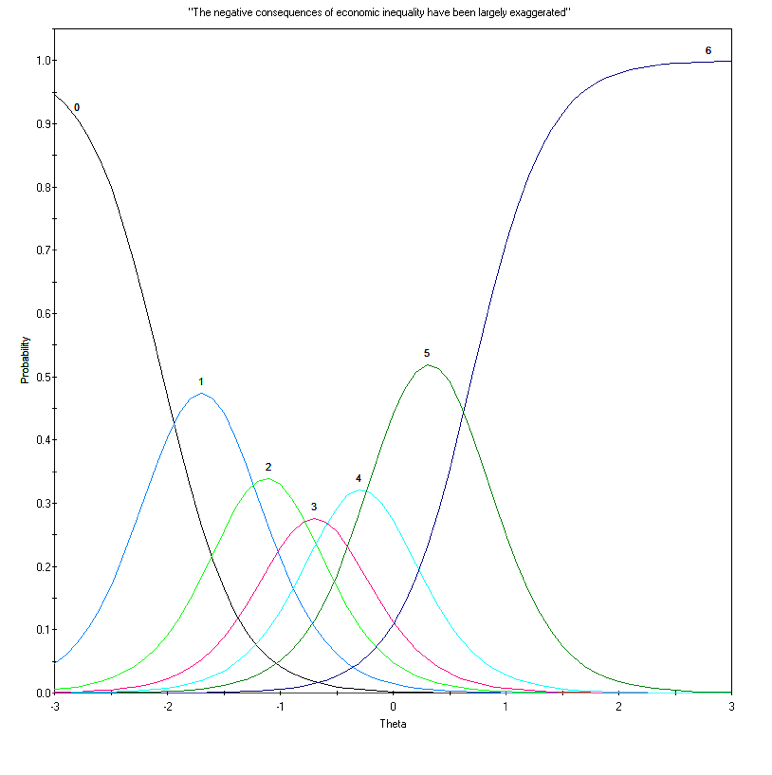

Supplement: S20 Fig — (PNG) [file pone.0218685.s020.png]

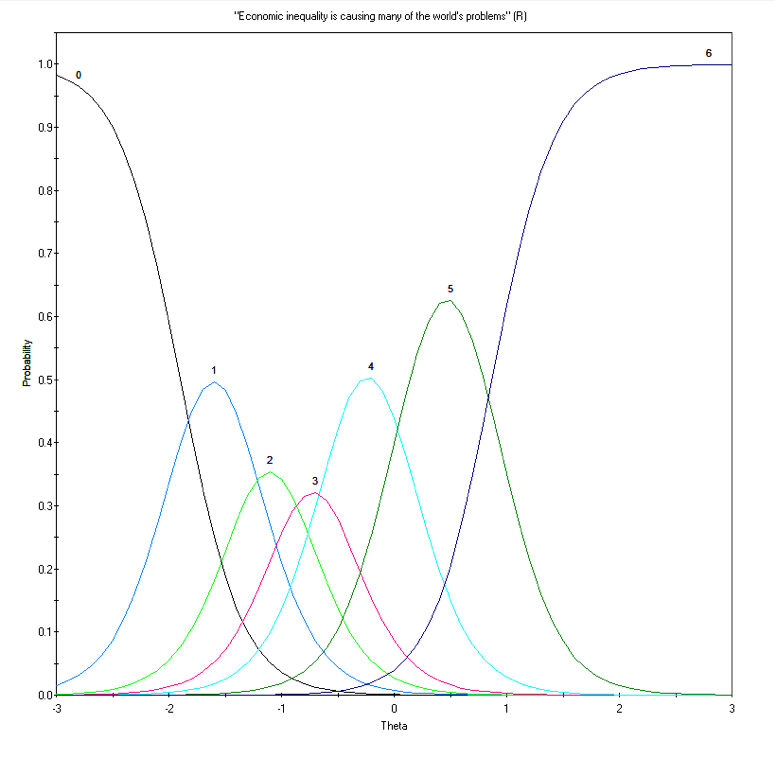

Supplement: S21 Fig — (PNG) [file pone.0218685.s021.png]

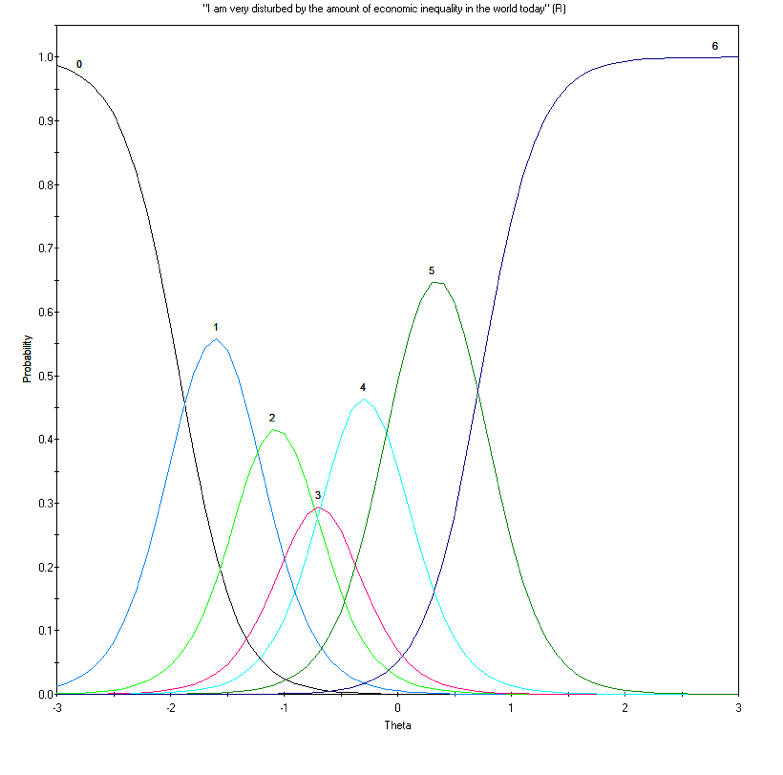

Supplement: S22 Fig — (PNG) [file pone.0218685.s022.png]

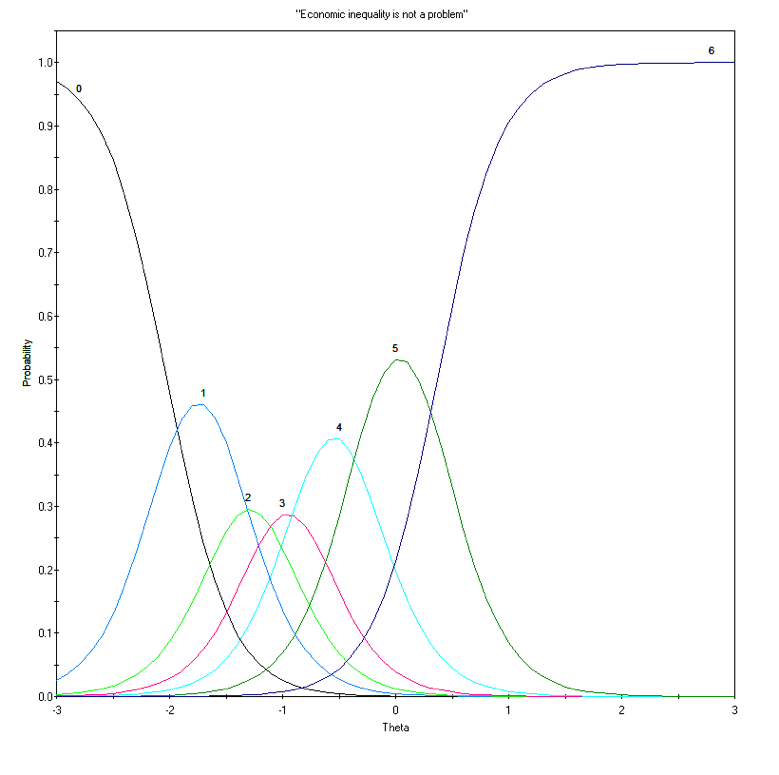

Supplement: S23 Fig — (PNG) [file pone.0218685.s023.png]

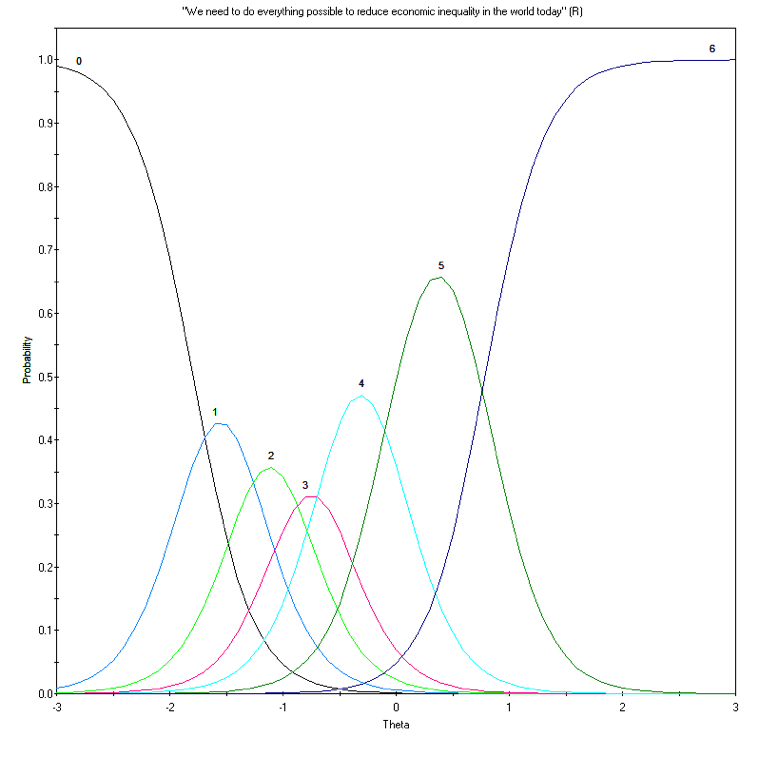

Supplement: S24 Fig — (PNG) [file pone.0218685.s024.png]

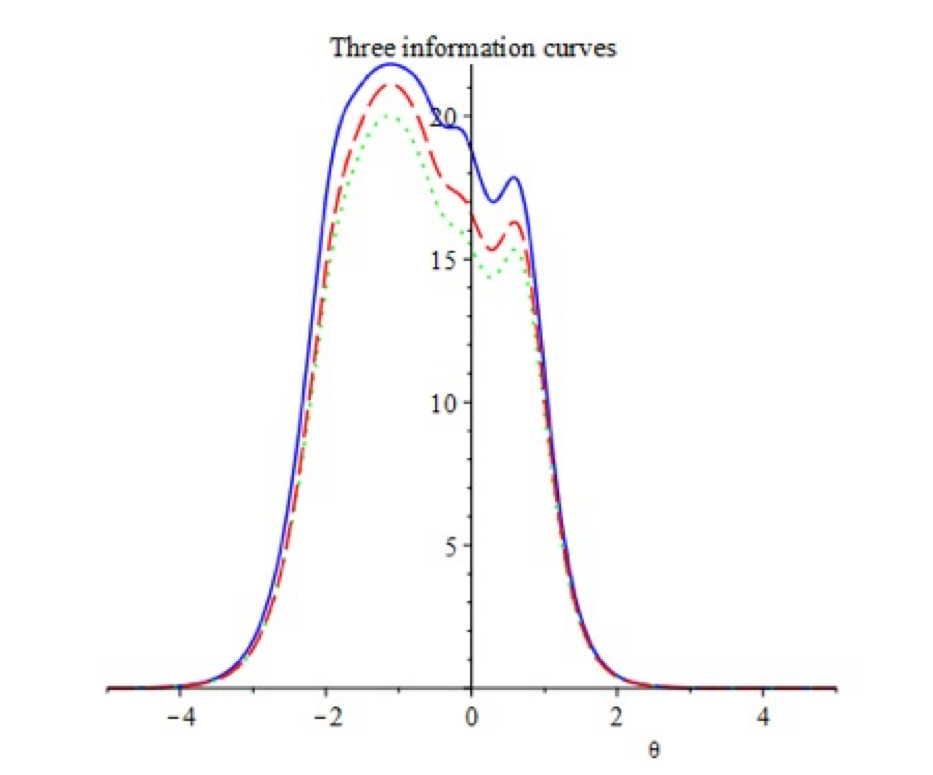

Supplement: S25 Fig — Note. The solid line is the Maximum Likelihood estimated theoretical maximum information, the dashed line is the aj weighted composite information, and the dotted line is the unit-weighted composite information. (PNG) [file pone.0218685.s025.png]
