# Supplementary material for: The Support for Economic Inequality Scale: Development and adjudication
Source: PLoS One. 2019 Jun 21;14(6):e0218685. doi: 10.1371/journal.pone.0218685 (PMC6588246; doi:10.1371/journal.pone.0218685)
Supplement: S1 Table — Note. Standard Errors for each parameter are in brackets, a is the item’s discrimination parameter, bs are the thresholds. (DOCX) [file pone.0218685.s026.docx]

**S1 Table. Graded Model Parameter Estimates for the full 18 items in Study 1.**

| Item | Discrimination (*a*) | Threshold 1 (*b*_1)_ | Threshold 2 (*b*_2)_ | Threshold 3 (*b*_3)_ | Threshold 4 (*b*_4)_ | Threshold 5 (*b*_5)_ | Threshold 6 (*b*_6)_ |
| --- | --- | --- | --- | --- | --- | --- | --- |
| 1 | 2.73 | -1.79 (.10) | -1.17 (.07) | -0.81 (.06) | -0.49 (.05) | 0.14 (.06) | 0.94 (.08) |
| 2 | 3.57 | -2.05 (.11) | -1.59 (.08) | -1.17 (.06) | -0.91 (.05) | -0.38 (.05) | 0.31 (.06) |
| 3 | 3.79 | -1.96 (.11) | -1.47 (.07) | -1.12 (.06) | -0.76 (.05) | -0.28 (.05) | 0.49 (.06) |
| 4 | 1.67 | -2.73 (.20) | -1.78 (.12) | -0.97 (.08) | -0.31 (.07) | 0.28 (.07) | 1.16 (.10) |
| 5 | 3.03 | -2.01 (.11) | -1.45 (.07) | -1.07 (.06) | -0.69 (.05) | 0.06 (.05) | 0.83 (.07) |
| 6 | 2.48 | -2.02 (.12) | -1.54 (.08) | -1.07 (.07) | -0.66 (.06) | 0.03 (.06) | 0.80 (.07) |
| 7 | 2.94 | -1.86 (.10) | -1.43 (.08) | -1.05 (.06) | -0.81 (.06) | -0.26 (.05) | 0.43 (.06) |
| 8 | 3.87 | -1.78 (.09) | -1.32 (.07) | -0.96 (.05) | -0.66 (.05) | -0.06 (.05) | 0.64 (.06) |
| 9 | 1.69 | -1.85 (.12) | -1.11 (.08) | -0.27 (.07) | 0.38 (.07) | 0.94 (.09) | 1.66 (.12) |
| 10 | 4.28 | -2.25 (.13) | -1.73 (.09) | -1.30 (.06) | -0.98 (.05) | -0.54 (.05) | 0.15 (.05) |
| 11 | 3.19 | -2.87 (.24) | -1.86 (.10) | -1.38 (.07) | -0.93 (.06) | -0.52 (.05) | 0.27 (.06) |
| 12 | 3.15 | -2.21 (.13) | -1.83 (.10) | -1.32 (.07) | -0.89 (.06) | -0.42 (.05) | 0.33 (.06) |
| 13 | 1.30 | -2.93 (.25) | -2.44 (.20) | -1.98 (.16) | -1.48 (.13) | -1.05 (.10) | -0.14 (.08) |
| 14 | 2.39 | -2.20 (.14) | -1.75 (.10) | -1.32 (.08) | -0.84 (.06) | -0.33 (.06) | 0.45 (.06) |
| 15 | 3.50 | -1.74 (.09) | -1.38 (.07) | -0.96 (.06) | -0.65 (.05) | -0.02 (.05) | 0.76 (.07) |
| 16 | 3.08 | -2.14 (.12) | -1.73 (.09) | -1.46 (.08) | -0.95 (.06) | -0.29 (.05) | 0.43 (.06) |
| 17 | 2.01 | -2.59 (.18) | -1.70 (.10) | -0.92 (.07) | -0.37 (.06) | 0.12 (.06) | 0.95 (.08) |
| 18 | 3.13 | -1.89 (.10) | -1.52 (.08) | -1.11 (.06) | -0.66 (.05) | -0.03 (.05) | 0.80 (.07) |

*Note*. Standard Errors for each parameter are in brackets, *a* is the item’s discrimination parameter, *bs* are the thresholds.
