# Supplementary material for: The Support for Economic Inequality Scale: Development and adjudication
Source: PLoS One. 2019 Jun 21;14(6):e0218685. doi: 10.1371/journal.pone.0218685 (PMC6588246; doi:10.1371/journal.pone.0218685)
Supplement: S2 Table — Note. Standard Errors for each parameter are in brackets. a is the item’s discrimination parameter, b are the thresholds. (DOCX) [file pone.0218685.s027.docx]

**S2 Table. Graded Model Item Parameter Estimates for the Final 5 Items in Study 1.**

| Item | *a* | *b*_1_ | *b*_2_ | *b*_3_ | *b*_4_ | *b*_5_ | *b*_6_ |
| --- | --- | --- | --- | --- | --- | --- | --- |
| 3 | 3.37 | -2.07 (.12) | -1.52 (.08) | -1.14 (.07) | -0.75 (.06) | -0.24 (.05) | 0.52 (.06) |
| 5 | 3.45 | -2.01 (.12) | -1.43 (.08) | -1.03 (.07) | -0.64 (.06) | 0.08 (.05) | 0.80 (.07) |
| 8 | 4.23 | -1.81 (.10) | -1.31 (.07) | -0.93 (.06) | -0.61 (.05) | -0.04 (.05) | 0.64 (.06) |
| 10 | 3.25 | -2.57 (.18) | -1.89 (.10) | -1.37 (.08) | -1.01 (.07) | -0.54 (.06) | 0.18 (.06) |
| 18 | 3.36 | -1.91 (.11) | -1.52 (.09) | -1.07 (.07) | -0.61 (.06) | 0.00 (.05) | 0.05 (.07) |

*Note.* Standard Errors for each parameter are in brackets. a is the item’s discrimination parameter, b are the thresholds.
