# Supplementary material for: The Support for Economic Inequality Scale: Development and adjudication
Source: PLoS One. 2019 Jun 21;14(6):e0218685. doi: 10.1371/journal.pone.0218685 (PMC6588246; doi:10.1371/journal.pone.0218685)
Supplement: S3 Table — (DOCX) [file pone.0218685.s028.docx]

**S3 Table. Item and Total Scale Information for the Final 5 Items in Study 1.**

| Item | Information | Proportion of  Total Information |
| --- | --- | --- |
| 3 | 8.40 | 14.5% |
| 5 | 13.41 | 23.2% |
| 8 | 13.63 | 23.6% |
| 10 | 12.65 | 21.9% |
| 18 | 9.73 | 16.8% |
| **Total** | **57.82** | **100%** |
