# Supplementary material for: The Support for Economic Inequality Scale: Development and adjudication
Source: PLoS One. 2019 Jun 21;14(6):e0218685. doi: 10.1371/journal.pone.0218685 (PMC6588246; doi:10.1371/journal.pone.0218685)
Supplement: S4 Table — (DOCX) [file pone.0218685.s029.docx]

**S4 Table. Goodness-of-fit Chi-Square tests for the 5 item scale in Study 2**

| Chi-Square | df | p-value | Chi-square/df |
| --- | --- | --- | --- |
| 142.62 | 75 | < .001 | 1.90 |
| 108.28 | 71 | .003 | 1.53 |
| 132.38 | 71 | < .001 | 1.86 |
| 159.82 | 82 | < .001 | 1.95 |
| 129.45 | 72 | < .001 | 1.80 |
