# Supplementary material for: The Support for Economic Inequality Scale: Development and adjudication
Source: PLoS One. 2019 Jun 21;14(6):e0218685. doi: 10.1371/journal.pone.0218685 (PMC6588246; doi:10.1371/journal.pone.0218685)
Supplement: S5 Table — (DOCX) [file pone.0218685.s030.docx]

**S5 Table. Goodness-of-fit Chi-Square tests for the 5 item scale in Study 3.**

| Chi-square | df | p-value | Chi-square/df |
| --- | --- | --- | --- |
| 108.18 | 76 | .009 | 1.42 |
| 98.73 | 62 | .002 | 1.59 |
| 136.23 | 63 | < .001 | 2.16 |
| 133.31 | 75 | < .001 | 1.78 |
| 113.83 | 67 | < .001 | 1.70 |
