# Supplementary material for: The Support for Economic Inequality Scale: Development and adjudication
Source: PLoS One. 2019 Jun 21;14(6):e0218685. doi: 10.1371/journal.pone.0218685 (PMC6588246; doi:10.1371/journal.pone.0218685)
Supplement: S6 Table — (DOCX) [file pone.0218685.s031.docx]

**S6 Table**. Correlations between all scales assessing convergent validity in Study 3.

|  | *1.* | *2.* | *3.* | *4.* | *5.* | *6.* | *7.* | *8.* | *9.* | *10.* | *11.* | *12.* | *13.* | *14.* |
| --- | --- | --- | --- | --- | --- | --- | --- | --- | --- | --- | --- | --- | --- | --- |
| 1. SEIS | -- |  |  |  |  |  |  |  |  |  |  |  |  |  |
| 2. GC | .59*** | -- |  |  |  |  |  |  |  |  |  |  |  |  |
| 3. SC | .53*** | .89*** | -- |  |  |  |  |  |  |  |  |  |  |  |
| 4. EC | .58*** | .88*** | .78*** | -- |  |  |  |  |  |  |  |  |  |  |
| 5. IU | .67*** | .51*** | .47*** | .50*** | -- |  |  |  |  |  |  |  |  |  |
| 6. PI | -.70*** | -.44*** | -.40*** | -.46*** | .51*** | -- |  |  |  |  |  |  |  |  |
| 7. SR | -.73*** | -.52*** | -.44*** | -.54*** | -.63*** | -.51*** | -- |  |  |  |  |  |  |  |
| 8. WG | -.18*** | -.18*** | -.15*** | -.19*** | -.13*** | .02 | .20*** | -- |  |  |  |  |  |  |
| 9. Comp | -.25*** | -.20*** | -.18*** | -.18*** | -.20*** | -.01** | .27*** | .30*** | -- |  |  |  |  |  |
| 10. Warm | -.31*** | -.21*** | -.21*** | -.20*** | -.25*** | -.15*** | .32*** | .24*** | .73*** | -- |  |  |  |  |
| 11. Emp | -.27*** | -.10* | -.21* | -.11** | -.28*** | -.32*** | .24*** | -.09* | -.01 | .19*** | -- |  |  |  |
| 12. PT | -.16*** | -.06 | -.06 | -.05 | -.14*** | -.04 | .20*** | .24*** | .23*** | .28*** | .38*** | -- |  |  |
| 13. BJW | .30*** | .29*** | .27*** | .30*** | .30*** | .40*** | -.23*** | .05 | .00 | -.01 | -.01 | .15*** | -- |  |
| 14. FW | .27*** | .27*** | .25*** | .24*** | .26*** | .28*** | -.23*** | -.14*** | -.13*** | -.09* | .07 | .09** | .51*** | -- |
| 15. Inc | .15*** | .08* | .06 | .16*** | .13*** | .11** | -.13** | .06 | -.14*** | -.11** | .03 | .03 | .20*** | .11** |

*Note.* SEIS = Support for Economic Inequality; GC = General Conservatism; SC = Social Conservatism; EC = Economic Conservatism; IU = Belief that Inequality is Unfixable; PI = Perceived Inequality; SR = Support for Redistribution; WG = Wealth Guilt; Comp = Perceptions of the poor as competent; Warm = Perceptions of the poor as warm; Emp = Empathy; PT = Prosocial Tendencies; BJW = Belief in a Just World; FW = Free Will; Inc = Income. * = *p* < .05, ** = *p* < .01, *** = *p* < .001
