# Supplementary material for: The Support for Economic Inequality Scale: Development and adjudication
Source: PLoS One. 2019 Jun 21;14(6):e0218685. doi: 10.1371/journal.pone.0218685 (PMC6588246; doi:10.1371/journal.pone.0218685)
Supplement: S7 Table — (DOCX) [file pone.0218685.s032.docx]

**S7 Table. Assessment of Multicollinearity in Study 5**

| Variable | Variance Inflation Factor | Klein’s Test for Multicollinearity |
| --- | --- | --- |
| Support for Economic Inequality | 4.87 | 1 |
| Belief in a Just World | 1.39 | 1 |
| Social Dominance Orientation | 3.14 | 1 |
| Economic System Justification | 4.35 | 1 |
| Protestant Work Ethic | 1.83 | 1 |
| Inegalitarianism | 3.26 | 1 |
| Support for Redistribution | 2.59 | 1 |
| WVS Support for Inequality | 2.54 | 1 |
| ISSP Support for Inequality | 2.58 | 1 |

*Note.* In Klein’s test for multicollinearity 1 means collinearity is detected in the test and 0 means collinearity was not detected in the test.
