# Supplementary material for: The Support for Economic Inequality Scale: Development and adjudication
Source: PLoS One. 2019 Jun 21;14(6):e0218685. doi: 10.1371/journal.pone.0218685 (PMC6588246; doi:10.1371/journal.pone.0218685)
Supplement: S8 Table — (DOCX) [file pone.0218685.s033.docx]

**S8 Table. Full SEM model output in Study 5**

Latent Variables:

| Estimate Std.Err z-value P(>\|z\|) Std.lv Std.all |
| --- |
| seis =~ |
| seis1 1.000 1.447 0.833 |
| seis2 1.018 0.038 26.811 0.000 1.474 0.854 |
| seis3 1.051 0.038 27.554 0.000 1.521 0.868 |
| seis4 0.918 0.043 21.471 0.000 1.329 0.736 |
| seis5 0.900 0.042 21.382 0.000 1.303 0.733 |
| bjw =~ |
| bjw1 1.000 0.702 0.665 |
| bjw2 0.797 0.064 12.368 0.000 0.559 0.520 |
| bjw3 1.076 0.066 16.295 0.000 0.755 0.705 |
| bjw4 1.072 0.065 16.550 0.000 0.752 0.717 |
| bjw5 0.823 0.058 14.078 0.000 0.578 0.598 |
| bjw6 0.613 0.055 11.059 0.000 0.430 0.461 |
| bjw7 0.999 0.060 16.640 0.000 0.701 0.722 |
| bjw8 0.838 0.064 13.180 0.000 0.588 0.557 |
| bjw9 1.028 0.067 15.429 0.000 0.722 0.663 |
| bjw10 0.989 0.058 16.973 0.000 0.694 0.738 |
| bjw11 1.068 0.067 16.031 0.000 0.749 0.692 |
| bjw12 0.714 0.054 13.143 0.000 0.501 0.555 |
| bjw13 0.970 0.064 15.154 0.000 0.681 0.649 |
| bjw14 0.809 0.062 13.150 0.000 0.568 0.555 |
| bjw15 1.104 0.069 15.986 0.000 0.775 0.690 |
| bjw16 1.051 0.062 17.022 0.000 0.738 0.741 |
| bjw17 0.996 0.060 16.480 0.000 0.699 0.714 |
| bjw18 0.960 0.056 17.017 0.000 0.674 0.741 |
| sdo =~ |
| sdo1 1.000 1.412 0.752 |
| sdo2 1.087 0.050 21.746 0.000 1.534 0.808 |
| sdo3 0.948 0.049 19.369 0.000 1.339 0.730 |
| sdo4 0.798 0.049 16.290 0.000 1.127 0.625 |
| sdo5 0.932 0.044 21.217 0.000 1.316 0.791 |
| sdo6 1.016 0.051 20.014 0.000 1.435 0.752 |
| sdo7 0.880 0.045 19.685 0.000 1.243 0.741 |
| sdo8 0.931 0.042 22.003 0.000 1.315 0.816 |
| sdo9 0.706 0.050 14.004 0.000 0.997 0.544 |
| sdo10 0.699 0.037 18.745 0.000 0.987 0.710 |
| sdo11 0.819 0.049 16.551 0.000 1.156 0.634 |
| sdo12 0.990 0.044 22.568 0.000 1.398 0.834 |
| sdo13 0.875 0.050 17.506 0.000 1.236 0.667 |
| sdo14 0.894 0.051 17.618 0.000 1.262 0.671 |
| sdo15 0.822 0.043 19.092 0.000 1.161 0.721 |
| ejst =~ |
| esjt1 1.000 1.255 0.574 |
| esjt2 0.283 0.064 4.452 0.000 0.355 0.182 |
| esjt3 1.076 0.084 12.742 0.000 1.351 0.603 |
| esjt4 0.928 0.073 12.645 0.000 1.164 0.596 |
| esjt5 1.188 0.090 13.156 0.000 1.491 0.630 |
| esjt6 0.705 0.073 9.602 0.000 0.885 0.421 |
| esjt7 1.303 0.089 14.623 0.000 1.636 0.738 |
| esjt8 1.096 0.088 12.500 0.000 1.375 0.587 |
| esjt9 1.077 0.085 12.666 0.000 1.351 0.598 |
| esjt10 1.083 0.084 12.929 0.000 1.359 0.615 |
| esjt11 0.798 0.078 10.168 0.000 1.001 0.451 |
| esjt12 1.240 0.088 14.165 0.000 1.556 0.702 |
| esjt13 0.567 0.073 7.728 0.000 0.712 0.328 |
| esjt14 1.430 0.098 14.636 0.000 1.794 0.739 |
| esjt15 1.308 0.092 14.209 0.000 1.642 0.706 |
| esjt16 1.524 0.099 15.345 0.000 1.912 0.797 |
| esjt17 0.762 0.079 9.613 0.000 0.957 0.422 |
| pwe =~ |
| pwe1 1.000 0.841 0.508 |
| pwe2 1.080 0.103 10.446 0.000 0.908 0.538 |
| pwe3 0.845 0.087 9.664 0.000 0.710 0.479 |
| pwe4 0.751 0.084 8.969 0.000 0.631 0.432 |
| pwe5 0.695 0.087 7.983 0.000 0.584 0.371 |
| pwe6 1.559 0.124 12.537 0.000 1.311 0.745 |
| pwe7 0.717 0.088 8.121 0.000 0.603 0.379 |
| pwe8 0.881 0.098 9.006 0.000 0.741 0.434 |
| pwe9 0.312 0.069 4.513 0.000 0.262 0.193 |
| pwe10 1.200 0.101 11.913 0.000 1.010 0.673 |
| pwe11 1.383 0.111 12.487 0.000 1.163 0.739 |
| pwe12 1.022 0.100 10.186 0.000 0.859 0.518 |
| pwe13 -0.384 0.082 -4.706 0.000 -0.323 -0.202 |
| pwe14 0.634 0.090 7.046 0.000 0.533 0.318 |
| pwe15 0.226 0.073 3.097 0.002 0.190 0.130 |
| pwe16 0.857 0.082 10.513 0.000 0.721 0.544 |
| pwe17 1.076 0.090 11.918 0.000 0.905 0.674 |
| pwe18 0.692 0.088 7.850 0.000 0.582 0.363 |
| pwe19 1.137 0.103 11.065 0.000 0.956 0.591 |
| inegal =~ |
| inegal1 1.000 1.030 0.807 |
| inegal2 0.874 0.040 21.929 0.000 0.900 0.765 |
| inegal3 0.968 0.044 21.889 0.000 0.997 0.764 |
| inegal4 1.035 0.045 23.131 0.000 1.066 0.796 |
| inegal5 0.889 0.044 20.410 0.000 0.916 0.725 |
| inegal6 1.121 0.044 25.294 0.000 1.155 0.848 |
| inegal7 1.116 0.047 23.873 0.000 1.149 0.814 |
| inegal8 0.001 0.011 0.108 0.914 0.001 0.004 |
|  |

Regressions:

| Estimate Std.Err z-value P(>\|z\|) Std.lv Std.all |
| --- |
| dict.give ~ |
| seis -0.746 0.262 -2.847 0.004 -1.079 -0.344 |
| bjw 0.472 0.244 1.940 0.052 0.332 0.106 |
| sdo 0.391 0.215 1.819 0.069 0.552 0.176 |
| ejst -0.783 0.488 -1.604 0.109 -0.982 -0.313 |
| pwe 0.423 0.330 1.282 0.200 0.356 0.113 |
| inegal 0.453 0.310 1.465 0.143 0.467 0.149 |

Covariances:

| Estimate Std.Err z-value P(>\|z\|) Std.lv Std.all |
| --- |
| seis ~~ |
| bjw 0.262 0.046 5.657 0.000 0.258 0.258 |
| sdo 1.729 0.131 13.158 0.000 0.846 0.846 |
| ejst 1.557 0.137 11.326 0.000 0.857 0.857 |
| pwe 0.550 0.070 7.858 0.000 0.452 0.452 |
| inegal 1.128 0.088 12.815 0.000 0.756 0.756 |
| bjw ~~ |
| sdo 0.199 0.043 4.567 0.000 0.200 0.200 |
| ejst 0.410 0.050 8.175 0.000 0.466 0.466 |
| pwe 0.353 0.041 8.624 0.000 0.598 0.598 |
| inegal 0.283 0.036 7.926 0.000 0.391 0.391 |
| sdo ~~ |
| ejst 1.454 0.134 10.851 0.000 0.820 0.820 |
| pwe 0.501 0.067 7.505 0.000 0.422 0.422 |
| inegal 1.057 0.087 12.131 0.000 0.726 0.726 |
| ejst ~~ |
| pwe 0.753 0.086 8.716 0.000 0.713 0.713 |
| inegal 1.130 0.100 11.290 0.000 0.874 0.874 |
| pwe ~~ |
| inegal 0.600 0.063 9.552 0.000 0.693 0.693 |

Variances:

| Estimate Std.Err z-value P(>\|z\|) Std.lv Std.all |
| --- |
| .seis1 0.924 0.061 15.039 0.000 0.924 0.306 |
| .seis2 0.809 0.056 14.478 0.000 0.809 0.271 |
| .seis3 0.755 0.054 13.968 0.000 0.755 0.246 |
| .seis4 1.498 0.091 16.483 0.000 1.498 0.459 |
| .seis5 1.459 0.088 16.503 0.000 1.459 0.462 |
| .bjw1 0.621 0.036 17.061 0.000 0.621 0.557 |
| .bjw2 0.845 0.048 17.570 0.000 0.845 0.730 |
| .bjw3 0.578 0.034 16.829 0.000 0.578 0.503 |
| .bjw4 0.534 0.032 16.741 0.000 0.534 0.485 |
| .bjw5 0.599 0.035 17.344 0.000 0.599 0.642 |
| .bjw6 0.684 0.039 17.690 0.000 0.684 0.787 |
| .bjw7 0.452 0.027 16.707 0.000 0.452 0.479 |
| .bjw8 0.769 0.044 17.475 0.000 0.769 0.690 |
| .bjw9 0.666 0.039 17.075 0.000 0.666 0.561 |
| .bjw10 0.402 0.024 16.573 0.000 0.402 0.455 |
| .bjw11 0.612 0.036 16.912 0.000 0.612 0.521 |
| .bjw12 0.564 0.032 17.480 0.000 0.564 0.692 |
| .bjw13 0.636 0.037 17.139 0.000 0.636 0.578 |
| .bjw14 0.723 0.041 17.479 0.000 0.723 0.691 |
| .bjw15 0.662 0.039 16.925 0.000 0.662 0.524 |
| .bjw16 0.447 0.027 16.551 0.000 0.447 0.451 |
| .bjw17 0.470 0.028 16.766 0.000 0.470 0.490 |
| .bjw18 0.374 0.023 16.554 0.000 0.374 0.451 |
| .sdo1 1.530 0.091 16.892 0.000 1.530 0.434 |
| .sdo2 1.254 0.076 16.396 0.000 1.254 0.348 |
| .sdo3 1.567 0.092 17.030 0.000 1.567 0.466 |
| .sdo4 1.979 0.113 17.462 0.000 1.979 0.609 |
| .sdo5 1.038 0.063 16.576 0.000 1.038 0.375 |
| .sdo6 1.584 0.094 16.895 0.000 1.584 0.435 |
| .sdo7 1.270 0.075 16.967 0.000 1.270 0.451 |
| .sdo8 0.868 0.053 16.297 0.000 0.868 0.334 |
| .sdo9 2.370 0.134 17.653 0.000 2.370 0.704 |
| .sdo10 0.961 0.056 17.142 0.000 0.961 0.496 |
| .sdo11 1.984 0.114 17.435 0.000 1.984 0.598 |
| .sdo12 0.857 0.053 16.048 0.000 0.857 0.305 |
| .sdo13 1.901 0.110 17.323 0.000 1.901 0.555 |
| .sdo14 1.941 0.112 17.309 0.000 1.941 0.549 |
| .sdo15 1.244 0.073 17.081 0.000 1.244 0.480 |
| .esjt1 3.200 0.182 17.539 0.000 3.200 0.670 |
| .esjt2 3.661 0.204 17.981 0.000 3.661 0.967 |
| .esjt3 3.197 0.183 17.463 0.000 3.197 0.637 |
| .esjt4 2.455 0.140 17.481 0.000 2.455 0.644 |
| .esjt5 3.372 0.194 17.376 0.000 3.372 0.603 |
| .esjt6 3.636 0.204 17.807 0.000 3.636 0.823 |
| .esjt7 2.243 0.133 16.851 0.000 2.243 0.456 |
| .esjt8 3.594 0.205 17.506 0.000 3.594 0.655 |
| .esjt9 3.283 0.188 17.477 0.000 3.283 0.643 |
| .esjt10 3.037 0.174 17.426 0.000 3.037 0.622 |
| .esjt11 3.929 0.221 17.768 0.000 3.929 0.797 |
| .esjt12 2.488 0.146 17.068 0.000 2.488 0.507 |
| .esjt13 4.192 0.234 17.898 0.000 4.192 0.892 |
| .esjt14 2.683 0.159 16.843 0.000 2.683 0.455 |
| .esjt15 2.720 0.160 17.050 0.000 2.720 0.502 |
| .esjt16 2.095 0.129 16.297 0.000 2.095 0.364 |
| .esjt17 4.235 0.238 17.806 0.000 4.235 0.822 |
| .pwe1 2.035 0.117 17.342 0.000 2.035 0.742 |
| .pwe2 2.020 0.117 17.224 0.000 2.020 0.710 |
| .pwe3 1.693 0.097 17.438 0.000 1.693 0.770 |
| .pwe4 1.741 0.099 17.571 0.000 1.741 0.814 |
| .pwe5 2.144 0.121 17.706 0.000 2.144 0.863 |
| .pwe6 1.378 0.088 15.590 0.000 1.378 0.445 |
| .pwe7 2.167 0.123 17.690 0.000 2.167 0.856 |
| .pwe8 2.362 0.134 17.565 0.000 2.362 0.812 |
| .pwe9 1.781 0.099 17.939 0.000 1.781 0.963 |
| .pwe10 1.229 0.075 16.405 0.000 1.229 0.547 |
| .pwe11 1.126 0.072 15.679 0.000 1.126 0.454 |
| .pwe12 2.014 0.116 17.305 0.000 2.014 0.732 |
| .pwe13 2.456 0.137 17.932 0.000 2.456 0.959 |
| .pwe14 2.529 0.142 17.797 0.000 2.529 0.899 |
| .pwe15 2.104 0.117 17.981 0.000 2.104 0.983 |
| .pwe16 1.237 0.072 17.202 0.000 1.237 0.704 |
| .pwe17 0.985 0.060 16.401 0.000 0.985 0.546 |
| .pwe18 2.232 0.126 17.721 0.000 2.232 0.868 |
| .pwe19 1.704 0.100 16.976 0.000 1.704 0.651 |
| .inegal1 0.570 0.036 15.761 0.000 0.570 0.349 |
| .inegal2 0.573 0.035 16.307 0.000 0.573 0.414 |
| .inegal3 0.707 0.043 16.318 0.000 0.707 0.416 |
| .inegal4 0.657 0.041 15.923 0.000 0.657 0.366 |
| .inegal5 0.757 0.045 16.680 0.000 0.757 0.475 |
| .inegal6 0.521 0.035 14.905 0.000 0.521 0.281 |
| .inegal7 0.672 0.043 15.632 0.000 0.672 0.337 |
| .inegal8 0.072 0.004 18.014 0.000 0.072 1.000 |
| .dict.give 8.848 0.506 17.499 0.000 8.848 0.897 |
| seis 2.095 0.163 12.845 0.000 1.000 1.000 |
| bjw 0.493 0.053 9.364 0.000 1.000 1.000 |
| sdo 1.994 0.179 11.139 0.000 1.000 1.000 |
| ejst 1.575 0.201 7.832 0.000 1.000 1.000 |
| pwe 0.707 0.107 6.618 0.000 1.000 1.000 |
| inegal 1.061 0.087 12.249 0.000 1.000 1.000 |
