# Supplementary material for: The Support for Economic Inequality Scale: Development and adjudication
Source: PLoS One. 2019 Jun 21;14(6):e0218685. doi: 10.1371/journal.pone.0218685 (PMC6588246; doi:10.1371/journal.pone.0218685)
Supplement: S9 Table — Note. Standard Errors for each parameter are in brackets. a is the item’s discrimination parameter, b are the thresholds. (DOCX) [file pone.0218685.s034.docx]

**S9 Table. Graded Model Item Parameter Estimates in low household income.**

| Item | *a* | *b*_1_ | *b*_2_ | *b*_3_ | *b*_4_ | *b*_5_ | *b*_6_ |
| --- | --- | --- | --- | --- | --- | --- | --- |
| 3 | 3.96 | -0.66 (.06) | 0.02 (.05) | 0.60 (.05) | 0.97 (.06) | 1.33 (.7) | 1.75 (.10) |
| 5 | 4.21 | -0.57 (.06) | 0.16 (.05) | 0.63 (.05) | 0.95 (.06) | 1.37 (.08) | 1.73 (.10) |
| 8 | 4.33 | -0.59 (.06) | 0.12 (.05) | 0.63 (.05) | 0.92 (.06) | 1.26 (.07) | 1.60 (.09) |
| 10 | 2.51 | -0.66 (.07) | 0.15 (.06) | 0.56 (.06) | 0.91 (.07) | 1.47 (.09) | 2.15 (.13) |
| 18 | 2.93 | -0.30 (.06) | 0.39 (.06) | 0.79 (.06) | 1.11 (.07) | 1.47 (.09) | 2.06 (.12) |

*Note.* Standard Errors for each parameter are in brackets. a is the item’s discrimination parameter, b are the thresholds.
