# Supplementary material for: The Support for Economic Inequality Scale: Development and adjudication
Source: PLoS One. 2019 Jun 21;14(6):e0218685. doi: 10.1371/journal.pone.0218685 (PMC6588246; doi:10.1371/journal.pone.0218685)
Supplement: S10 Table — (DOCX) [file pone.0218685.s035.docx]

**S10 Table. Item and Total Scale Information for low household income**

| Item | Information | Proportion of  Total Information |
| --- | --- | --- |
| 3 | 12.12 | 20.9% |
| 5 | 13.56 | 23.3% |
| 8 | 13.49 | 23.2% |
| 10 | 8.40 | 14.5% |
| 18 | 10.56 | 18.2% |
| **Total** | **58.12** | **100%** |
