# Supplementary material for: The Support for Economic Inequality Scale: Development and adjudication
Source: PLoS One. 2019 Jun 21;14(6):e0218685. doi: 10.1371/journal.pone.0218685 (PMC6588246; doi:10.1371/journal.pone.0218685)
Supplement: S11 Table — Note. Standard Errors for each parameter are in brackets. a is the item’s discrimination parameter, b are the thresholds. (DOCX) [file pone.0218685.s036.docx]

**S11 Table. Graded Model Item Parameter Estimates in high household income.**

| Item | *a* | *b*_1_ | *b*_2_ | *b*_3_ | *b*_4_ | *b*_5_ | *b*_6_ |
| --- | --- | --- | --- | --- | --- | --- | --- |
| 3 | 2.97 | -1.75 (.15) | -0.73 (.08) | 0.04 (.05) | 0.42 (.06) | 0.91 (.09) | 1.81 (.18) |
| 5 | 3.49 | -1.60 (.14) | -0.66 (.08) | 0.04 (.05) | 0.43 (.06) | 0.84 (.08) | 1.81 (.17) |
| 8 | 3.19 | -1.68 (.15) | -0.67 (.08) | 0.04 (.05) | 0.46 (.06) | 0.93 (.09) | 1.75 (.16) |
| 10 | 2.38 | -1.58 (.15) | -0.56 (.08) | -0.03 (.06) | 0.43 (.07) | 1.02 (.10) | 1.92 (.18) |
| 18 | 2.57 | -1.13 (.11) | -0.23 (.06) | 0.35 (.06) | 0.72 (.09) | 1.20 (.12) | 2.02 (.20) |

*Note.* Standard Errors for each parameter are in brackets. a is the item’s discrimination parameter, b are the thresholds.
