# Supplementary material for: The Support for Economic Inequality Scale: Development and adjudication
Source: PLoS One. 2019 Jun 21;14(6):e0218685. doi: 10.1371/journal.pone.0218685 (PMC6588246; doi:10.1371/journal.pone.0218685)
Supplement: S12 Table — (DOCX) [file pone.0218685.s037.docx]

**S12 Table. Item and Total Scale Information for high household income**

| Item | Information | Proportion of  Total Information |
| --- | --- | --- |
| 3 | 12.06 | 19.8% |
| 5 | 15.28 | 25.0% |
| 8 | 14.87 | 24.4% |
| 10 | 9.59 | 15.7% |
| 18 | 9.24 | 15.1% |
| **Total** | **61.04** | **100%** |
