# Supplementary material for: The Support for Economic Inequality Scale: Development and adjudication
Source: PLoS One. 2019 Jun 21;14(6):e0218685. doi: 10.1371/journal.pone.0218685 (PMC6588246; doi:10.1371/journal.pone.0218685)
Supplement: S13 Table — (DOCX) [file pone.0218685.s038.docx]

**S13 Table. Item and Total Scale Information for Democrats and Republicans**

|  | Democrats | | Republicans | |
| --- | --- | --- | --- | --- |
| Item | Information | Proportion of  Total Information | Information | Proportion of  Total Information |
| 3 | 12.38 | 21.6% | 11.65 | 23.1% |
| 5 | 13.98 | 24.4% | 14.17 | 28.1% |
| 8 | 13.51 | 23.5% | 12.72 | 25.2% |
| 10 | 8.99 | 15.7% | 5.21 | 10.3% |
| 18 | 8.54 | 14.9% | 6.64 | 13.2% |
| **Total** | **57.41** | **100%** | **50.38** | **100%** |
