# Supplementary material for: The Support for Economic Inequality Scale: Development and adjudication
Source: PLoS One. 2019 Jun 21;14(6):e0218685. doi: 10.1371/journal.pone.0218685 (PMC6588246; doi:10.1371/journal.pone.0218685)
Supplement: S14 Table — (DOCX) [file pone.0218685.s039.docx]

**S14 Table. Goodness-of-fit Chi-Square tests for the five-item scale for Democrats and Republicans**

|  | Democrats | | | | Republicans | | | |
| --- | --- | --- | --- | --- | --- | --- | --- | --- |
| Item | Chi-square | df | p-value | Chi-square/df | Chi-Square | df | p-value | Chi-square/df |
| 3 | 95.88 | 50 | <.001 | 1.92 | 130.46 | 65 | <.001 | 2.01 |
| 5 | 55.43 | 49 | .24 | 1.13 | 100.24 | 63 | .002 | 1.59 |
| 8 | 73.93 | 50 | .02 | 1.48 | 102.90 | 61 | < .001 | 1.69 |
| 10 | 96.39 | 60 | <.001 | 1.61 | 149.66 | 84 | < .001 | 1.78 |
| 18 | 71.94 | 55 | .06 | 1.31 | 161.10 | 78 | < .001 | 2.07 |
