# Supplementary material for: The Support for Economic Inequality Scale: Development and adjudication
Source: PLoS One. 2019 Jun 21;14(6):e0218685. doi: 10.1371/journal.pone.0218685 (PMC6588246; doi:10.1371/journal.pone.0218685)
Supplement: S15 Table — (DOCX) [file pone.0218685.s040.docx]

**S15 Table. Goodness-of-fit Chi-Square tests for the five-item scale in low and high household income**

|  | Low Household Income | | | | High Household Income | | | |
| --- | --- | --- | --- | --- | --- | --- | --- | --- |
| Item | Chi-square | df | p-value | Chi-square/df | Chi-Square | df | p-value | Chi-square/df |
| 3 | 179.93 | 73 | <.001 | 2.46 | 129.10 | 72 | <.001 | 1.79 |
| 5 | 123.13 | 70 | <.001 | 1.76 | 106.55 | 69 | .002 | 1.54 |
| 8 | 117.64 | 70 | <.001 | 1.68 | 135.14 | 70 | < .001 | 1.93 |
| 10 | 170.93 | 88 | <.001 | 1.94 | 151.05 | 81 | < .001 | 1.86 |
| 18 | 164.94 | 84 | <.001 | 1.96 | 154.86 | 79 | < .001 | 1.96 |
